# Reference-Based RADseq Unravels the Evolutionary History of Polar Species in ‘the Crux Lichenologorum’ Genus *Usnea* (Parmeliaceae, Ascomycota)

**DOI:** 10.3390/jof9010099

**Published:** 2023-01-11

**Authors:** Ana Otero, Alejandrina Barcenas-Peña, H. Thorsten Lumbsch, Felix Grewe

**Affiliations:** The Grainger Bioinformatics Center & Negaunee Integrative Research Center, Science & Education, The Field Museum, Chicago, IL 60605, USA

**Keywords:** next-generation sequencing, lichen-forming fungi, phylogenomics, systematics, species delimitation, species complex

## Abstract

Nearly 90% of fungal diversity, one of the most speciose branches in the tree of life, remains undescribed. Lichenized fungi as symbiotic associations are still a challenge for species delimitation, and current species diversity is vastly underestimated. The ongoing democratization of Next-Generation Sequencing is turning the tables. Particularly, reference-based RADseq allows for metagenomic filtering of the symbiont sequence and yields robust phylogenomic trees of closely related species. We implemented reference-based RADseq to disentangle the evolution of neuropogonoid lichens, which inhabit harsh environments and belong to *Usnea* (Parmeliaceae, Ascomycota), one of the most taxonomically intriguing genera within lichenized fungi. Full taxon coverage of neuropogonoid lichens was sampled for the first time, coupled with phenotype characterizations. More than 20,000 loci of 126 specimens were analyzed through concatenated and coalescent-based methods, including time calibrations. Our analysis addressed the major taxonomic discussions over recent decades. Subsequently, two species are newly described, namely *U. aymondiana* and *U. fibriloides*, and three species names are resurrected. The late Miocene and Pliocene-Pleistocene boundary is inferred as the timeframe for neuropogonoid lichen diversification. Ultimately, this study helped fill the gap of fungal diversity by setting a solid backbone phylogeny which raises new questions about which factors may trigger complex evolutionary scenarios.

## 1. Introduction

The kingdom Fungi represents one of the most diverse branches in the tree of life [1]. Naturalists have pointed mainly to the vast diversity of growth forms, reproductive strategies, habitats, biotic associations, and lifestyles that fungi can show or be adapted to. Unfortunately, all this complexity has often hindered understanding their place in the tree of life and underestimated their species diversity [2]. The latest estimates indicated between 2.2 and 3.8 million species of fungi, from which only 148,000 (the majority in Ascomycota) are currently accepted [3,4]. Thus, nearly 90% of fungi diversity remains unknown, versus 50% for better-studied groups, such as plants [5]. One of the major reasons for hidden fungal diversity is poorly explored habitats, such as lichenicolous fungi [4]. Another reason is the restudy of known taxa through cutting-edge molecular methods.

Lichenized fungi are spread along the Fungal Tree of Life with more than 16,000 species, mostly belonging to Ascomycota [6]. The extraordinary nature of lichens as symbiotic associations involving multiple kingdoms and complex hierarchies of living organisms (e.g., primary and secondary fungi, algae/cyanobacteria, and microbiome elements) arises as a major challenge for species delimitation [7]. Indeed, traditional phenotype-based taxonomy (i.e., morphological and chemical traits) has led to a vast underestimation of actual species diversity [6].

The integration of molecular methods for species delimitation has revolutionized lichen systematics [8,9,10]. From the early use of single barcoding loci, such as ITS, to the Next-Generation Sequencing (NGS) tools, the systematics of lichenized fungi has benefited, and a wide range of complex evolutionary processes hidden by morphology are now coming to light [11]. This new knowledge through molecular studies contributes to an increase in the number of described species. New lineages are inferred as a result of three main processes: (1) homoplasy (i.e., similar traits have evolved independently and multiple times along the phylogeny) is perhaps the most frequently inferred, and one of the major flaws of a phenotypic-only-based taxonomy [12,13]; (2) so-called cryptic species (i.e., species with no observable and apparent phenotypic differences) are being abundantly argued for many newly described species [14,15,16]; (3) species with apparent phenotypic differences showed short genetic distances, often interpreted as a result of recent diversification and incomplete lineage sorting [17,18] or as different morphotypes of the same species [19,20,21]. This complex scenario of lichen speciation and the exceptionally high infraspecific phenotypic plasticity reduce the efficacy of some widely used barcode genes [22,23,24]; thus, genome-wide sequencing approaches are required to build robust phylogenies [25,26].

Among the various NGS techniques developed over the past decades, restriction site-associated sequencing (RADseq) is one of the most successful and cost-effective tools for species delimitation. In RADseq, high-throughput sequencing generates data from thousands of loci over the genome that elucidates complex speciation questions [27]. For lichenized fungi, using reference-based RADseq (i.e., loci are mapped back to a reference genome) allows for the exclusion of loci from other organisms occurring in these symbioses so that robust phylogenomic trees can be generated among closely related species [28,29,30]. Nevertheless, the assembly of massive amounts of genomic loci with potentially conflicting phylogenetic signals entails new challenges and trade-offs between the computational capacity and the accuracy to integrate complex biological scenarios such as ancient introgression, hybridization, or incomplete lineage sorting into the bioinformatic pipelines [31]. Fortunately, the rapid proliferation of workflows for NGS data that accomplish these possible biases is parallel to the popularization of these sequencing methods [32,33]. Ultimately, incorporating high-throughput sequencing as a tool for species delimitation is helping to assess the taxonomic value of phenotypical characters traditionally described, which may have been considered taxonomically irrelevant under a plethora of morphological varieties often found in lichens [6].

Neuropogonoid lichens comprise around 17 species of lichen-forming fungi belonging to the most iconic beard-like macrolichen: the genus *Usnea* Dill. ex Adans., currently considered among the ten most speciose genera (~350 spp, Parmeliaceae, Ascomycota; [34]). Neuropogonoid species are distributed in polar regions and high-alpine regions. They are adapted to harsh microclimates, including extreme temperature variation, high radiation and winds, and scarce water availability [35]. Contrary to the most widely repeated pattern of higher species richness towards the equator line [36], this group has its main center of diversity in Antarctica and subantarctic regions [35]. Recently, these organisms were noted as global warming bioindicators since their habitats are documented to be some of the most sensitive to temperature increases [37,38,39,40,41]. Diagnostic characters for this group are the saxicolous habit, strong black pigmentation of the thallus (often variegated) and apothecia discs, and a relatively thick cortex [42]. Usnic acid is present in the cortex of all species, and varied combinations of depsides and depsidones in the medulla are described to characterize different races within species, including an abundance of acid-deficient lineages [35]. These markedly distinguishing morphological and chemical traits are often associated with the adaptation to harsh environments [42] and have led lichenologists to consider this group either as a subgenus [35,43,44,45,46], a section [47], or a separate genus [48,49,50,51,52,53,54,55]. Lastly, multi-gene phylogenies revealed the group as polyphyletic within *Usnea* subgen. *Usnea* and the name ‘neuropogonoid’ were coined to refer to the *Neuropogon* core group [56]. The systematics of *Usnea* at a specific level is considered exceptionally difficult, often known as ‘the crux lichenologorum’ by modern lichenologists, and neuropogonoid lichens follow this pattern with several species delimitations [57]. Although phylogenetic studies based on a few loci have unveiled new clades of neuropogonoid lichens (e.g., *U. lambii, U. messutiae, U. pallidocarpa*, and *U. ushuaiensis*; [42,58,59]), these studies have not been able to disentangle phylogenetic relationships among these lineages and species boundaries of some groups remain unclear [34]. RADseq has already shed light on an example of the widely debated species-pairs for *U. antarctica* and *U. aurantiacoatra* [60]. Still, questions remain for systematics and the spatio-temporal evolutionary framework that shaped the diversification and distribution of neuropogonoid lichens.

In this study, we aim to keep filling the gap of fungal diversity by using cutting-edge methods such as reference-based RAD sequencing to investigate the phylogenomic relationships and species boundaries for the 17 species of neuropogonoid lichens in combination with morphological and chemical characters to circumscribe these clades. To this end, we considered the following specific objectives: (1) to reconstruct the backbone phylogeny for neuropogonoid lichens; (2) to infer the evolutionary timeframe for the neuropogonoid clade; and (3) to contribute to fungal taxonomy by addressing the taxonomic rearrangements inferred from the phylogenetic findings.

## 2. Materials and Methods

### 2.1. Specimen Sampling

Taxon sampling of neuropogonoid lichens was based on the taxa validly described at the species level in [35], which constitutes the most comprehensive monograph of the neuropogonoid group to date. Thirteen of the fifteen species recognized by Walker [35] were included in the present study (Table S1, in Supporting Information). The two species not considered for the present study were: (1) *U. durietzii* Motyka, interpreted as intermediate between the neuropogonoid group and sect. *Usnea* [35], which was subsequently shown to be only distantly related to neuropogonoid lichens [56]; and (2) *U. neuropogonoides* Motyka, described from few and immature specimens with unclear relationships to neuropogonoid lichens [35]. Four additional species recently accepted were also included here (i.e., *U. lambii, U. messutiae*, [42]; *U. ushuaiensis*, [58]; and *U. pallidocarpa* [59]). In total, the ingroup comprises 17 currently accepted species. In addition, we propose below the acceptance of five additional species. A total of 120 samples from neuropogonoid lichens were studied, plus six samples of three other *Usnea* species used as the outgroup. Samples were obtained from multiple field campaigns performed by Phylogenomic Initiative Centre at The Field Museum as well as from herbaria collections, including The Field Museum (F), Herbarium Senckenbergianum (FR), and external research collaborations (Table S1).

### 2.2. Morphological and Chemical Characterization

**Figure 1:**
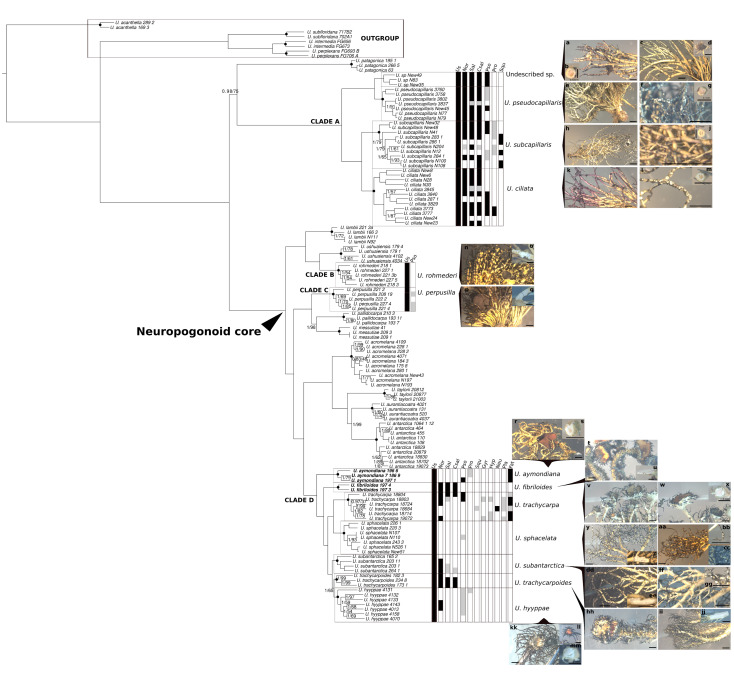
Phylogenetic reconstruction of neuropogonoid lichens based on concatenated DNA sequences of 21,831 loci (m4 dataset) obtained from Bayesian inference (BI) through the ExaBayes software. Values at nodes indicate posterior probability yielded by BI on the left and bootstrap support (BS) from maximum-likelihood (ML) inference (RaxML-NG software) on the right. Maximum support for both inferences (i.e., BI = 1; BS = 100) was obtained for those nodes with no values. Dark circles on the nodes represent the crown of the monophyletic species considered. Clades of taxonomic interest are marked with letters A-D. Chemical characters are shown for clades A-D. Up to 12 chemical acids were found: Us, Usnic acid; Nor, norstictic acid; Sal, salazinic acid; Csal, consalazinic acid; Pso, psoromic acid; Pro, protocetraric acid; Squ, squamatic acid; Gyr, gyrophoric acid; Hyp, hypostrepsilic acid; Neu, neuropogolic acid; Pla, placodolic acid; Fat, fatty acids. High concentration of the substance is indicated with black bands, low concentration or traces are indicated in gray bands, and non-colored bands indicate the absence of the substance. Images of the thallus, reproductive structures and internal structure are shown for representative samples from each species at Clades A-D: (**a**) *U. sp* New49; (**b**) *U. sp* New35, thallus cross section; (**c**) *U. sp* New35, basis; (**d**) *U. sp* New35, apothecia; (**e**) *U. pseudocapillaris* 3760; (**f**) *U. pseudocapillaris* 3758, soredia; (**g**) *U. pseudocapillaris* 3760, thallus cross section; (**h**) *U. subcapillaris* New 32; (**i**) *U. subcapillaris* N100; (**j**) *U. subcapillaris* New 32, thallus cross section; (**k**) *U. ciliata* New 23, (**l**) *U. ciliata* New 6; (**m**) *U. ciliata* N27, thallus cross section; (**n**) *U. rohmederi* 221-3B; (**o**) *U. rohmederi* 218-1, thallus longitudinal section; (**p**) *U. perpusilla* 222-2; (**q**) *U. perpusilla* 208-19, thallus longitudinal section (cortex is indicated in red, medulla in blue, axis in black); (**r**) *U. aymondiana* 197-1; (**s**) *U. aymondiana* 196-6, thallus cross section; (**t**) *U. fibriloides* 197-3; (**u**) *U. fibriloides* 197-3, thallus longitudinal section (cortex is indicated in red, medulla in blue, axis in black); (**v**) *U. trachycarpa* 19072; (**w**) *U. trachycarpa* 19072, apothecia; (**x**) *U. trachycarpa* 18684, thallus cross section; (**y**) *U. sphacelata* 220-3; (**z**) *U. sphacelata* 226-1, thallus longitudinal section; (**aa**) *U. sphacelata* 226-1; (**bb**) *U. sphacelata* N110, soredia; (**cc**) *U. sphacelata* 220-3, soredia; (**dd**) *U. subantarctica* 203-11; (**ee**) *U. subantarctica* 203-11, thallus longitudinal section; (**ff**) *U. subantarctica* 203-11; (**gg**) *U. subantarctica* 203-11, soredia; (**hh**) *U. trachycarpoides* 173-1; (**ii**) *U. trachycarpoides* 234-8; (**jj**) *U. trachycarpoides* 182-3, thallus longitudinal section; (**kk**) *U. hyyppae* 4070; (**ll**) *U. hyyppae* 4158; (**mm**) *U. hyyppae* 4158, thallus cross section. All black scales indicate 2 mm except for those where specific number is shown. More information about specimen vouchers is provided in Table S1 of the Supplementary Material.

The morphological and chemical investigation was performed for the taxa belonging to the four selected clades with a particular taxonomic interest: (1) Clade A: New Zealand endemic species, (2) Clade B: *U. rohmederi*, (3) Clade C: *U. perpusilla*, and (4) Clade D: *U. trachycarpa* complex (see Figure 1). Morphological features were examined using a stereo-microscope Olympus SZX-ILLD100 (Olympus Co., Tokyo, Japan), focusing primarily on ten morphological characters that were considered valuable for species delimitation by Walker (1985, [35]): (1) habit, (2) holdfast branching, (3) branching pattern above the holdfast (4) thallus pigmentation, (5) surface ornamentation of the thallus, (6) presence of papillae, (7) presence of fibrils, (8) internal structure, (9) presence of apothecia, and (10) presence of vegetative propagules (see Table S2). High-performance thin-layer chromatography (HPTLC) was done for the same species of taxonomic interest. Accordingly, a total of 73 samples were soaked in acetone overnight and chromatographed using solvent system C [61,62,63,64]. Major and minor substances were identified, and chemosyndromes for all species were assigned based on the previous chemosyndrome characterization [65].

### 2.3. DNA Extraction and RAD Library Preparation

DNA extraction and RAD libraries were conducted through the Next-Generation Facility at the University of Wisconsin Biotechnology Center (UWBC). DNA extraction was performed using the DNeasy mericon Food Kit (Qiagen, Hilden, Germany). Paired-end RADseq libraries were prepared using restriction enzyme ApeKI following [66] and sequenced on a Novaseq6000 Illumina Inc. (San Diego, CA, USA) at UWBC by setting one million reads per sample. Processed data were returned in the FASTQ format, with Phred quality scores for all bases.

### 2.4. RADseq Assembly

Forward reads of all samples were processed in ipyrad v.0.9.84 [67] using the High-Performance Computing (HPC) cluster installed at The Grainger Bioinformatics Center (The Field Museum). Ipyrad was run using the “reference” assembly method, which maps RAD sequences to a reference genome to determine homology. The genome of the lichen-fungal culture of *U. hakonensis* Asahina [68] was used as the reference. The mapping sorted the metagenomic lichen sequences for reads derived from the lichen fungus. Unmapped sequences that did not match the lichen-fungal reference were discarded (reference-based method, [67]). Ploidy was set to one to reflect the haploid nature of the fungal thallus. The sensitivity of the results regarding data matrix completeness and the number of loci was assessed by testing three different values of minimum taxon coverage per locus (i.e., parameter ‘m’): (1) the default value, m = 4; (2) 25% of the total samples, m = 32; and (3) 50% of the total samples, m = 65. Thus, a total of three assembled data sets were produced and denoted as m4, m25, and m50, respectively.

### 2.5. Phylogenomic Analyses

The resulting matrix of concatenated loci for the three datasets was used as the input to perform phylogenomic analyses. Maximum likelihood (ML) inference was performed through RAxML-Next-Generation v.1.1. [69] by running the ‘all-in-one’ option (i.e., ML tree search + bootstrapping including both Felsenstein Bootstrapping and Transfer Bootstrap Expectation method) over 20 different starting trees, GTR + GAMMA model and using the automatic bootstrap stopping parameter with the majority rule criterion (autoMRE, cutoff: 0.030000). Bootstrap supports are represented on the best-scored ML tree. This software re-implements former algorithms already integrated in RAxML/ExaML and provides new features that outperform the accuracy, flexibility, speed, and scalability making it more suitable for large empirical datasets [69]. Additionally, in order to assess the performance of this recently developed method, RAxML v8.2.12 [70] (rapid bootstrapping, GTRGAMMA model, autoMRE) was also implemented for the three datasets. The matrix producing the highest average bootstrap support was also analyzed using Bayesian inference (BI) in ExaBayes 1.5.1 [71]. Bayesian inference was set for two runs, four coupled chains, and one million generations with a sampling frequency of 500 and a burn-in proportion of 0.25. A coalescent-based method for phylogenomic inference was also implemented for the three data matrices through SVDquartets [72] in PAUP [73]. Individuals were grouped according to current species circumscriptions, and all possible quartets were evaluated with 100 bootstrap replicates.

Potential historical introgression between the early diverging lineages of *U. patagonica* and the clade of the neuropogonoid core was tested through four-taxon D-statistic (ABBA-BABA) tests [74]. In a pectinate four-taxon tree [(((P1,P2),P3),P4)], where A and B represent ancestral and derived alleles, respectively, the P3 taxon is expected to share derived alleles with either of the two sister species, P1 (BABA) or P2 (ABBA) in the same proportion. An excess of BABA or ABBA pattern is interpreted as evidence of ancient admixture between lineages. Accordingly, using the loci matrix for m4 dataset as the input, *U. patagonica* samples were set up as the P1 taxon. A selection of two samples (i.e., the two with the highest number of loci in the m4 dataset) per species from clade A were set up as the P2 taxon. Likewise, species of the neuropogonoid core were set to represent the P3 taxon. Ultimately, samples from the outgroup were assigned to the P4 taxon. We implemented *D*-statistic calculations using allele frequencies through the ipa.baba module of ipyrad, specifically designed for RADseq data sets. Tests were run for all possible sample combinations in a four-taxon tree (P1–P4). Significance was assessed through 1000 bootstrap replicates for each test, and Z-score was calculated to measure the number of bootstrap standard deviations in which the *D*-statistic deviates from zero. Significant patterns were considered for a Z-score > 3, which correspond to a conservative cutoff α = 0.01 [75] after the Benjamini–Hochberg correction [76].

### 2.6. Estimates of Divergence Times

A penalized likelihood method, implemented in TreePL [77], was used to estimate a time-calibrated tree. TreePL is an upgraded version of r8s [78] that accounts for among-branch rate heterogeneity by applying the so-called smoothing parameter [79]. This method relies on branch-length estimates from prior ML or BI analyses and node age constraints to estimate a different substitution rate for each branch using stochastic optimization and hill-climbing gradient-based methods. The analysis is less time-consuming and is suitable for dealing with large amounts of data with high percentages of missing data, such as RADseq [31,80,81,82]. Taxon sampling for divergence time estimation was reduced to include a single sample per species by choosing the samples with the highest number of loci retrieved from the three datasets (Table S3). Then, the ipyrad assembly branch from the full matrix (m4) was run from step 7, and RaxML analysis was performed on the reduced matrix (i.e., one tip per species) following the same parameter settings as for the full matrix. Two calibration points were set based on the highest posterior density intervals yielded by [83] for the genus *Usnea*: (1) root age (minimum age = 17.1355; maximum age = 29.2712) and (2) crown node of neuropogonoid lichens (ingroup) (minimum age = 4.5719; maximum age = 14.7433). A first analysis using the “prime” option was run to select the optimal set of parameter values. Accordingly, a second run was performed by setting the following optimal parameters: gradient-based (opt) optimizer = 1; autodifferentiation-based (optad) = 2; autodifferentiation cross-validation-based optimizers (optcvad) = 2. In this second run, random subsample and replicate cross-validation (RSRCV) was set to identify the best value for the smoothing parameter (lambda). The best chi-square value for the smoothing parameter (lambda = 1e-8) was implemented for the third and final analysis. For the three runs, the thorough analysis option, 200,000 penalized likelihood iterations, and 5000 cross-validation iterations were set. To account for uncertainty in branch lengths due to variance in molecular substitution across the RADseq loci, TreePL was run for each of the bootstrap trees obtained from the Maximum likelihood analyses following [27]. The maximum clade credibility method implemented in TreeAnnotator [84] was used to build the consensus-calibrated tree.

Ultimately, caution and sensitiveness are needed when interpreting divergence time estimates from RAD-seq data. We here accounted for some of the main concerns argued [85] by: (1) including a broad range of the loci rather than maximizing the number of individuals sampled per locus (i.e., m4 dataset is used), (2) providing more than one calibration point, and (3) accounting for the branch-length uncertainty due to variance in molecular substitution across the RADseq loci by running the TreePL on every bootstrap tree generated in the ML reconstruction (see above).

## 3. Results

### 3.1. Morphological and Chemical Characterization

Morphological characterization was performed for the four clades of particular taxonomical interest (Clades A–D; Figure 1, Table S2).

Clade A is characterized by a smooth, waxy surface of the thallus, a yellowish thallus, blackened toward the tips, variegated in *U. subcapillaris* and violaceous grading in *U. ciliata* that also has black-edge annulations. The habit and mode of the branching varied from an erect, proliferating holdfast and moderately branched in *U. ciliata* to a pendulous/subpendulous, delimited holdfast and extensively branched in *U. pseudocapillaris* and *U. subcapillaris* (Figure 1c–k). Thick cortices distinguished the subclade of *U. subcapillaris* and *U. ciliata* from the clade of *U. pseudocapillaris* with thinner cortices. A wide axis (more than half of the thallus width) was generally found in the taxa of clade A except in *U. ciliata* with narrower axes. Placement and ornamentation of apothecia distinguished *U. ciliata* (subterminal, short excipular rays) from *U. subcapillaris* (lateral, long excipular rays). Soredia found in *U. pseudocapillaris* are plane, rounded to confluent or occasionally minute and punctiform. Otherwise, among mostly apotheciate specimens, soredia are also found in *U. ciliata*, which are plane, rounded, and black-spotted (Figure 1k–m). An undescribed taxon is found as the fertile counterpart of *U. pseudocapillaris* (Figure 1a–d), which showed some traits similar to those found in *U. ciliata*, such as erect, proliferating holdfast, black-edge annulations, and terminal apothecia. However, the pigmentation pattern and internal structure are similar to the sorediate sister clade *U. pseudocapillaris*.

Morphological characters also support the recognition of *U. rohmederi* at the specific rank (currently in *U. perpusilla*) in agreement with the RADseq phylogenomic reconstructions (see below). Thus, *U. rohmederi* (clade B) is distinguished from *U. perpusilla* s.str. (clade C) by a delimited holdfast, black variegated pigmentation toward the apices with frequent long excipular rays in the apothecia that are often variegated. Meanwhile, *U. perpusilla* has a richly proliferating holdfast, barely variegated pigmentation, and lacks excipular rays in the apothecia (Figure 1n–q).

Morphological characterization of clade D resulted in the recognition of seven species, including two species newly described below in congruence with the phylogenomic reconstructions (Figure 1r–mm). Brownish to red apothecia discs distinguished the two early diverging, newly described species (*U. aymondiana* and *U. fibriloides*) from the remaining apotheciate taxa in clade D. In turn, *U. fibriloides* is distinguished from *U. aymondiana* by the presence of numerous minute black fibrils along both thallus and the apothecial discs (Figure 1t–u). Papillae in *U. aymondiana* are abundant but less dense, and fibrils are larger than in *U. fibriloides* (Figure 1r–s). Besides, *U. aymondiana* has a narrower axis width than *U. fibriloides* (Figure 1r–u). The clade of *U. trachycarpa* was confined to specimens from the Kerguelen, where the type was described. Specimens from other regions were found to belong to other species. *Usnea trachycarpa* has a delimited holdfast that is moderately branched above, with a yellowish to black tips pigmentation and numerous black pigmented papillae and fibrils variable in length. This species is distinguished from other apotheciate-related taxa by having a wide axis, compact medulla, cupular and flat apothecial discs, orange and black pigmented, often ornamented with papillae and short fibrils (Figure 1w). *Usnea trachycarpoides* and *U. hyyppae* showed similar traits as found in *U. trachycarpa* but differed in having a sublax medulla, narrower axis, more flattened, orange and terminal apothecia, and longer fibrils. *Usnea trachycarpoides* is variable in the density of papillae from absent to abundant (Figure 1hh–jj, Table S2). Two species (*U. sphacelata* and *U. subantarctica*) were found to be sorediate in clade D. *Usnea sphacelata* is distinguished from *U. subantarctica* by a smooth thallus surface with spread papillae on main branches and moderately branching above the holdfast, rare fibrils, and a thin cortex, with a sublax axis (Figure 1y–cc, Table S2). Soredia in both species are globose and also excavate in *U. sphacelata.*

A total of 11 medullary substances and five chemosyndromes already described for *Usnea* [65] were identified through the HPTLC (Table 1 and Table S4): (1) the hypostrepsilic acid chemosyndrome (constituted by the dibenzofurans isostrepsilic acid and hypostrepsilic acid) and (2) the neuropogolic acid chemosyndrome (constituted by the aliphatic compounds proto- and neuropogolic acids). The hypostrepsilic acid and neuropogolic acid chemosyndromes were detected in low concentrations only for *U. trachycarpa.* Other identified chemosyndromes were (3) the fumarprotocetraric syndrome that is here represented by the protocetraric acid detected in low concentrations for *U. subcapillaris*, *U. ciliata*, and *U. aymondiana*, (4) the salazinic acid chemosyndrome (including salazinic/consalazinic and norstictic acids) that was frequently present as major substance in clade A, and finally, (5) the psoromic acid chemosyndrome (including psoromic and 2’-*O*-de-methylpsoromic acids) that was sporadically present along the four clades as accessory substance except for some lineages in clade A in which it constituted a major substance (Figure 1).

Within-species chemical variability was moderate (1-2 chemotypes) except for *U. ciliata, U. subcapillaris*, and *U. trachycarpa*, which had up to six different chemotypes (Table S4). Usnic acid was found in the cortex as the major substance in all species examined. Clade A, comprising endemic species from New Zealand (*U. ciliata*, *U. subcapillaris*, *U. pseudocapillaris*), had similar chemical patterns in which the salazinic acid chemosyndrome was present in most chemotypes (Table 1 and Table S4). *Usnea subcapillaris*, the most chemically diverse species of this clade, distinguished from the other species by the presence of squamatic acid as one of the major substances for some samples. *Usnea rohmederi* (Clade B) and *U. perpusilla* (Clade C) usually lacked medullary substances, but some specimens of *U. perpusilla* contained traces of psoromic acid (Table 1 and Table S4). High chemical variability was observed for species of clade D, with *U. trachycarpa* as the most chemically diverse (6 chemotypes). The two newly described species differ in their chemistry. While *U. aymondiana* contains fatty acids, sparse psoromic acid, and traces of protocetraric acid, *U. fibriloides* contains the salazinic acid chemosyndrome. Among the other apotheciate species, *U. trachycarpa* showed the highest variability containing chemotypes assigned up to four chemosyndromes. *Usnea trachycarpoides* contains the salazinic acid chemosyndrome, whereas *U. hyyppae* only had norstictic acid occasionally in two samples and traces of psoromic and protocetraric acids. Regarding the two sorediate species of this clade, *U. sphacelata* was medullary deficient with only traces of psoromic acid in one of the specimens, whereas *U. subantarctica* showed two chemotypes including salazinic acid chemosyndrome.

### 3.2. Assembly of RAD Sequencing

Three matrices of 126 samples were obtained after the ipyrad assembly, filtering, and processing of all reads sequenced. As a result, the total numbers of (1) loci filtered ranged from 21,831 (m4) to 4504 (m50), (2) single nucleotide polymorphisms (snps) from 381,659 (m4) to 166,242 (m50), and (3) missing data percentage from 82.91% (m4) to 72.41% (m50) (Table 2).

### 3.3. Phylogenomic Analyses and Divergence Times Estimation

The topologies inferred through RAxML-NG (ML) using the full matrix of concatenated RADseq loci were congruent among the three parameter settings tested, and only minor differences in bootstrap supports (BS) were observed (Figure S1a). All species were retrieved as monophyletic with maximum BS. Similarly, high BS was inferred for all phylogenetic relationships among neuropogonoid species except for *U. patagonica*-clade A with moderate supports (BS = 65–84), *U. trachycarpoides*-*U. hyyppae* (BS = 44–65), and *U. taylorii-U. aurantiacoatra-U. antarctica* with BS = 75 for m50 dataset (Figure S1a). The highest overall BS average was yielded by m4 dataset that was therefore selected for Bayesian inference (BI, Figure 1). In the BI analysis, maximum values of posterior probability (PP) confirmed the monophyly of neuropogonoid species. Overall, PP values for all nodes ranged from 0.97 to 1, except for the clade of *U. acromelana* (PP = 0.83) (Figure 1). Topologies of ML with RAxML-NG and BI were congruent, and only minor node support differences were retrieved (Figure 1). Thus, *U. patagonica* is inferred as sister to clade A which comprised three endemic species from Australasia: (1) *U. pseudocapillaris*, sister to a clade including (2) *U. ciliata* and (3) *U. subcapillaris*. The neuropogonoid core is formed by an early diverging clade comprising *U. lambii* as sister to a clade including *U. ushuaiensis* and *U. rohmederi* (clade B, Figure 1) and a major clade comprising three subclades: (1) *U. perpusilla* (clade C) sister to *U. messutiae*-*U. pallidocarpa*; (2) *U. acromelana*, *U. taylorii* and *U. aurantiacoatra-U. antarctica*; and (3) Clade D, including *U. aymondiana* and *U. fibriloides* as early diverging branches and a subclade formed by *U. trachycarpa*-*U. sphacelata* and *U. subantarctica*, *U. trachycarpoides-U. hyppae*. Only one incongruence was found for the ML inference from RAxML v8.2.12 that retrieved *U. patagonica* as sister to the Neuropogonoid core (Figure S1b). This position contrasts with the topologies yielded by all the other phylogenomic approaches herein implemented where *U. patagonica* is sister to clade A (i.e., RAxML-NG v.1.1., ExaBayes and SVDquartets).

**Figure 2:**
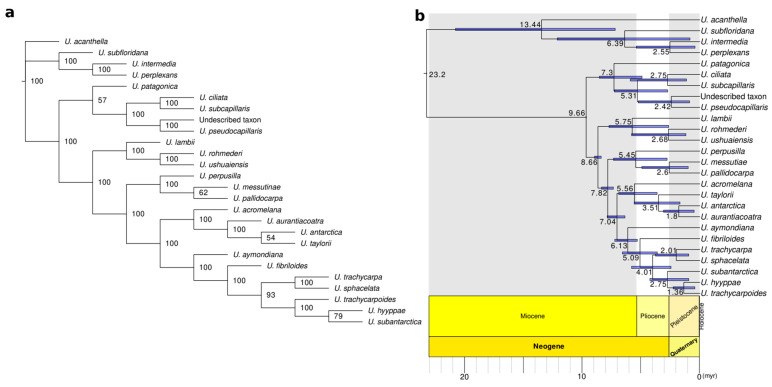
(**a**) SVDquartets species tree of neuropogonoid lichens for m4 dataset. BS are indicated for each node.; (**b**) Time-calibrated phylogeny obtained from treePL by using maximum clade credibility from bootstrap trees of the maximum-likelihood analysis of the m4 of reduced sampling matrix. Bootstrap supports (BS) for all branches are 100 except for the subclade *U. pallidocarpa*–*U. messutiae* with BS = 92. Mean ages in millions of years (myr) are indicated for each node. Node bars in blue indicate the node age ranges taking into account the branch length variance along the bootstrap trees. The international stratigraphic scale is included from 23 myr until the present.

The coalescent-based analyses using the SVDquartets also resulted in overall high BS values (Figure 2a and Figure S2). Coalescent-based topologies agreed with the BI and RAxML-NG retrieving *U. patagonica* as sister to clade A, however with lower BS values. (BS = 45–58; Figure 2a and Figure S2). Other differences in the coalescent-based topologies were a low-supported sister relationship between *U. subantarctica* and *U. hyyppae* (BS = 77–84; Figure 2a and Figure S2) and a poorly supported sister relationship of *U. taylorii* and *U. antarctica* to *U. aurantiacoatra* (BS = 48–54; Figure 2a and Figure S2).

The ML phylogenomic trees resulting from the reduced matrix (one sample per species) yielded overall high BS support for all phylogenetic relationships, and the topologies of the trees were congruent with coalescent-based trees, BI and RAxML-NG tree for the relationship between *U. patagonica* and Clade A (Figure S3).

Introgression analysis resulted in a total of 55,275 tests generated from all possible sample combinations for a four-taxon tree [(((P1,P2),P3),P4)] (Figure S4). BABA pattern indicating introgression between *U. patagonica* and the neuropogonoid core. It was the most frequent in 97% of all the 1636 tests that resulted in significance with a Z-score ≥ 3 (Table S5). An average of 2501.85 loci per test were included (Table S5).

The divergence time estimation (Figure 2b) indicated that most species (14 out of 21) originated either in the Pliocene-Pleistocene boundary (~3 myr) or early Pleistocene (~1.5 myr) (Table 3). The earliest diverging species, *U. patagonica* was estimated to have evolved during the late Miocene and Miocene-Pliocene boundary, around 7.3 myr ago (4.91–8.54 Bootstrapped variance, BV).

### 3.4. Taxonomy

#### 3.4.1. *Usnea aymondiana* A.Otero, Barcenas-Peña, Lumbsch & Grewe sp. nov. (Figure 1r,s)

MycoBank: MB846765

Diagnosis: *Usnea aymondiana* is distinguished from *U. trachycarpa* s.l. by having brown to dark red apothecia discs and it is distinguished in turn from its closest relative, *U. fibriloides* by having a more richly branched thallus, less dense papillae, larger fibrils in both branches and apothecial discs and fatty acid compounds.

Type: Argentina, Santa Cruz Province, Mount Aymond, 52°07′ S, 69°31′ W, 12.2003, N. Wirtz & M.I. Messuti 7-186-9, Herbarium code: C0172407F (BCRU—holotype; F—isotype).

Etymology: The epithet refers to the locality where the type was collected, Monte Aymond in Santa Cruz Province, Argentina.

Description: Thallus approx. 2–4 cm tall, arising from a delimited unpigmented holdfast. Erect thallus moderately branched with yellowish main branches that become extensively ramified and black pigmented toward the tips. Side branches mostly variegated with bands of black pigment towards the tips. Rugose thallus surface usually foveolate with abundant yellow papillae in the main branches and short fibrils (c. 1 mm) variegated or continuously black-pigmented on all branches. Cortex thick, medulla sublax, axis compact less than half or half of the branch diameter. Soredia and isidiomorphs not seen. Apothecia frequent, subterminal, with brownish-red discs, margin verrucose with short black fibrils and frequent long yellow or black variegated excipular rays. Pycnidia not seen.

Chemistry: HPTLC: Usnic acid is always present, and fumarprotocetraric acid and psoromic acid chemosyndromes are frequent or sometimes absent (Elix et al., 2007, [65]). Chemotypes: (1) Major substance: usnic acid; minor substance: psoromic acid; (2): Major substance: usnic acid; minor substance: ± protocetraric acid. Fatty acids found in both chemotypes.

Distribution: Only known from a single locality in Mount Aymond at 200 m altitude, close to Rio Gallegos (Santa Cruz province), at the border between Argentina and Chile.

Notes: The new species is distinguished from the other *U. trachycarpa* allies by having brown to dark red apothecia discs and can be readily distinguished from *U. fibriloides* by a more richly branched thallus, less dense papillae, the presence of larger fibrils in both branches and apothecial discs and also chemical differences (see below).

Additional specimens examined: ARGENTINA. Santa Cruz Province, Mount Aymond, 52°07′ S, 69°31′ W, 12/.003, N. Wirtz & M.I. Messuti 196-6 (F). Santa Cruz Province, Mount Aymond, 52°07′ S, 69°31′ W, 12.2003, N. Wirtz & M.I. Messuti 197-1 (F).

#### 3.4.2. *Usnea fibriloides* A.Otero, Barcenas-Peña, Lumbsch & Grewe sp. nov. (Figure 1t,u)

MycoBank: MB846792

Diagnosis: *Usnea fibriloides* is distinguished from *U. trachycarpa* s.l. by having a barely branched and unpigmented thallus covered by minute thick, black-pigmented fibrils and densely minute fibrillated apothecial margins without excipular rays. *Usnea fibriloides* also differs from its closest relative, *U. aymondiana* by the presence of norstictic and salazinic chemosyndromes.

Type: Argentina, Santa Cruz Province, Mount Aymond, 52°07′ S, 69°31′ W, 12.2003, N. Wirtz & M.I. Messuti, 197-3. Herbarium code: C0172408F (BCRU—holotype, F—isotype).

Etymology: The epithet refers to the presence of distinguishing minute black pigmented fibrils all along the thallus and at the margins of apothecial discs.

Description: Thallus approx. 3–4 cm tall, arising from a delimited unpigmented holdfast. Erect thallus barely branched, unpigmented, and rarely ramified. The thallus surface highly rugose and densely papillae, abundantly covered by minute thick, black-pigmented fibrils (less than 1 mm). Cortex thick, medulla sublax, axis compact half of the branch diameter. Soredia and isidiomorphs not seen. Apothecia frequent, terminal, brownish-red disc, margin verrucose and abundant minute black fibrils, without excipular rays. Pycnidia not seen.

Chemistry: HPTLC: Major substance: usnic acid; minor substances or traces: salazinic acid, consalazinic acid, norstictic acid (Elix et al., 2007, [65]).

Distribution: Only known from a single locality in Mount Aymond at 200 m altitude, close to Rio Gallegos (Santa Cruz province), at the border between Argentina and Chile.

Notes: The new species is distinguished from the other *U. trachycarpa* allies by having a barely branched and unpigmented thallus covered by minute thick, black-pigmented fibrils and densely minute fibrillated apothecial margins without excipular rays. Differences in secondary metabolites are also found with its closest relative *U. aymondiana* (see above).

Additional specimens examined: ARGENTINA. Santa Cruz Province, Mount Aymond, 52°07′ S, 69°31′ W, 12.2003, N. Wirtz & M.I. Messuti 197-4 (F).

## 4. Discussion

The phylogenetic structure of neuropogonoid lichens has been debated in the literature [34]. For decades since the first phenotype-based reviews, lichenologists have discussed not only possible phylogenetic relationships among these lichens but also their evolutionary timeframe, including their biogeographical history [35,48,86]. Nevertheless, in most cases, either the lack of a complete sampling or the insufficient variability of genetic markers has masked a robust reconstruction of the evolutionary history [34]. For the first time, we present a complete phylogenomic representation of the neuropogonoid group using cutting-edge sequencing methods and NGS assembly tools. The reference-based RAD sequencing method resulted in a robust phylogeny that unveils the evolutionary history of this group of lichenized fungi (Figure 1). These phylogenomic results have enabled us to resolve two species complexes: the *U. perpusilla*-complex and *U. trachycarpa*-complex. Subsequently, five additional names are proposed for the neuropogonoid lichens, including three species names resurrected and two newly described species. Moreover, two species are sequenced here for the first time: *U. pseudocapillaris* and *U. taylorii*, thus resulting in a full taxon coverage of this group [34].

Neuropogonoid lichens were supported as a monophyletic group in agreement with previous phylogenies [34,42,56]. All phylogenomic trees retrieved monophyly for all the species within the neuropogonoid group and highly supported phylogenetic relationships among lineages (Figure 1). Similarly, the coalescent-based approach resulted in a congruent topology with maximum support for most phylogenetic relationships, only differing in lower BS support for for three nodes: (1) early divergence of *U. patagonica* as sister to clade A and shallow divergence within (2) the *U. taylorii-U. antarctica-U. aurantiacoatra* clade, and (3) *U. hyyppae-U. subantarctica*. Incomplete lineage sorting (ILS) or ancient introgression can be masked under high bootstrap supports when analyzing large-scale, concatenated datasets. These effects can be driven by only a few loci [87,88,89]. Coalescent-based methods are shown to integrate and overcome the conflicting signal derived from these processes. In particular, SVDquartets is expected to be confidently applicable for empirical systems where levels of gene flow were maintained time after the speciation [90]. Furthermore, methods such as BABA-ABBA tests enable distinguishing between a more stochastic signal of ILS from the directional asymmetry of shared ancestral loci in ancient introgression events among closely related species [74,89,91]. Our introgression tests suggested a significative signal for ancient introgression between *U. patagonica* and the core of neuropogonoid lichens (Figure S4, Table S5) that was also reflected by the coalescent-based tree (Figure 2a) and the incongruence retrieved between ExaBayes and RAxML-NG, versus RaxML v.8. concatenated-based trees (Figure 1 and Figure S1). Otherwise, further investigations on the most recent divergences of *U. taylorii-U. antarctica-U. aurantiacoatra* clade and *U. hyyppae-U. subantarctica* are needed to disentangle potential ILS or ongoing gene flow already suggested by the moderate support of these nodes for coalescent-based reconstructions.

### 4.1. Contributions to the Systematics of Neuropogonoid Lichens

Circumscriptions of some of the major species of neuropogonoid lichens [35] mostly agree with our phylogenetic reconstructions. Nevertheless, the information provided by more than 20,000 loci yielded by the high performance of RAD sequencing allowed us to disentangle some of the most complicated species aggregates [58]. Here, we discuss the contributions to the systematics of neuropogonoid lichens by the RAD sequencing analysis.

### 4.2. The Usnea Perpusilla Complex

The *Usnea perpusilla* complex exhibits an array of variable morphological traits represented by both fertile and asexual taxa with a smooth, faveolate, waxy, epapillate and extensively black-pigmented surface combined with a thin axis and a lax medulla [35,58]. Wirtz et al. (2008, [58]) addressed this complex through a cohesion approach of species delimitation resulting in the recognition of five species: (1) *U. lambii*, (2) *U. pallidocarpa*, (3) *U. perpusilla*, (4) *U. messutiae*, and (5) *U. ushuaiensis*. Nevertheless, limitations of the data set prevented them from addressing the phylogenetic relationship among species. Our study revealed the presence of a sixth lineage for which we propose to resurrect the name *U. rohmederi* (I.M. Lamb) I.M. Lamb [92], previously regarded as a synonym of *U. perpusilla* [35]. Our phylogenomic results suggest that the *U. perpusilla* complex is divided into two independent lineages: (1) an early diverging clade formed by *U. lambii* sister to a clade including *U. ushuaiensis* and *U. rohmederi*, and (2) a derived clade formed by *U. perpusilla* sister to the clade of *U. messutiae* and *U. pallidocarpa* (Figure 1). *Usnea perpusilla* and *U. rohmederi* overlap in their distributional ranges from Tierra del Fuego to higher latitudes in Argentina and morphologically, *U. rohmederi* is distinguished by the presence of a more delimited holdfast, black variegated pigmentation toward the apices and frequent long, variegated excipular rays (Figure 1n–q, Table S2).

### 4.3. The Usnea Trachycarpa Complex

The *U. trachycarpa* complex includes species exhibiting a wide range of fertile phenotypes and closely related asexual lineages [56]. The abundant presence of fibrils, papillate thallus, and rufous-brown apothecial discs with numerous excipular rays characterize this group [35,48]. Chemically, various chemosyndromes of β-orcinol depsidones are found as medullary substances [35]. The extensive phenotypical variation around the diagnostic traits has led to the description of a plethora of varieties, subspecies, forms, and even different species often associated with particular geographic distributions [35]. Some authors regarded this variability as part of the intraspecific variation (e.g., [35,48]). Furthermore, the asexual species *U. subantarctica* has been considered as the sterile counterpart of *U. trachycarpa* (e.g., [35]), which was subsequently supported based on the paraphyly of *U. trachycarpa* (e.g., [34,42,56,93]). The combination of full taxon coverage of this species complex and large-scale genomic data resulted in a revised species delimitation. We here propose to accept seven distinct species: (1) *U. aymondiana* and (2) *U. fibriloides*, two newly described species currently only known from Mt. Aymond (Patagonia, Argentina); (3) *U. hyppae* Räsänen [94] and (4) *U. trachycarpoides* (Vain.) C.W. Dodge [95], two resurrected species names previously synonymized with *U. trachycarpa* [35]; and the traditionally accepted species (5) *U. trachycarpa*, (6) *U. subantarctica*, and (7) *U. sphacelata* (Clade D, Figure 1). The seven species have been congruently inferred as well supported monophyletic lineages and are also supported by morphological and chemical characters (Figure 1, Table 1, Tables S2 and S4). The two newly described species are inferred as early diverging branches of this clade and are characterized by phenotypical differences, such as darker brownish to red apothecia unlike more orange apothecia for the other apotheciate lineages in this clade. Besides, *U. fibriloides* is readily distinguished by numerous particularly short and thick fibrils all along the thallus and apothecia that give it a distinguishing aspect (Figure 1t). Contrasting chemistry is found between the two new species with norstistic, salazinic and consalazinic acids in *U. fibriloides*, whereas *U. aymondiana* only contained psoromic acid and traces of protocetraric acid (Figure 1, Table 1 and Table S4). *Usnea trachycarpoides* and *U. hyyppae* occur in southern Chile and formed a well-supported clade together with the sorediate *U. subantarctica*. Maximum likelihood and Bayesian trees inferred a sister relationship between the two apotheciate species although with moderate support under ML (BS = 65; Figure 1), whereas coalescent-based methods revealed *U. hyyppae* as sister to the sorediate *U. subantarctica* with higher support (BS = 77–83, Figure 2 and Figure S2). Our results suggest that *U. hyyppae* is likely the fertile counterpart of *U. subantarctica.* However, further studies including more thorough sampling in this area will be necessary to test our hypothesis. Otherwise, RADseq phylogenies indicate that *U. trachycarpa* is restricted to the Kerguelen Islands from where the type was described [96]. The bipolar sorediate species *U. sphacelata* is strongly supported as sister species to *U. trachycarpa*, thus pointing to a new case in which the asexual sister clade would show a much wider distribution range than the fertile sister clade [60].

### 4.4. The New Zealand Clade

Around 28 species of *Usnea* are known to occur in New Zealand [97]. However, this apparently low number of species is thought to be highly underestimated as a result of the lack of regional studies and the complicated taxonomy of this group [97]. Our study supports the presence of six species of neuropogonoid *Usnea* taxa in New Zealand [35]: *U. acromelana, U. ciliata*, *U. lambii*, *U. pseudocapillaris* (sequenced here for the first time)*, U. sphacelata* and *U. subcapillaris*. *Usnea antarctica* has been recorded in New Zealand, but our molecular study does not support the presence of this species in the country. Furthermore, *U. lambii*, separated from *U. sphacelata* [42] is here recorded and sequenced in New Zealand for the first time (Figure 1). Three species that appear to be endemic to New Zealand (*U. ciliata*, *U. pseudocapillaris*, and *U. subcapillaris*) formed one of the early diverging clades of neuropogonoid lichens (Clade A, Figure 1). *Usnea ciliata* and *U. subcapillaris*, two fertile species, have a sister group relationship and form a sister group to the sorediate *U. pseudocapillaris*, discarding *U. subcapillaris* as its species pair as suggested by [35]. Interestingly, the fertile counterpart of *U. pseudocapillaris* (herein referred as *undescribed taxon*) is inferred as the intermediate phenotype of *U. ciliata* and *U. pseudocapillaris* (Figure 1a–d, Table 1, Tables S2 and S4). Further investigations on this putative new taxon are necessary before a formal description. For the three other species, independent colonizations of New Zealand during the Pleistocene of asexual species are most likely (see *U. lambii*, *U. acromelana*, and *U. sphacelata*, Figure 1). Thus, we here unveil multiple and independent origins of neuropogonoid lichens from New Zealand in contrast to previous hypotheses which suggested a unique monophyletic clade for all New Zealand neuropogonoid taxa, questioning an amphi-Pacific disjunction within some species (e.g., *U. acromelana*; [34]).

### 4.5. A Spatio-Temporal Evolutionary Framework of Neuropogonoid Lichens

The Miocene and Pliocene have been the main periods for the divergence of major lineages within parmelioid lichens (e.g., *Flavoparmelia*, [98]; *Melanohalea*, [99]; *Montanelia*, [100]; *Xanthoparmelia*, [101]) including the crown age of *Usnea* [83,102]. The progressive global cooling since the Oligocene-Miocene boundary [103], together with the increase in the aridity, promoted a transition to temperate forests and ultimately to more open habitats [104,105] that seem to have triggered the diversity of Parmeliaceae [101]. Neuropogonoid species are exceptionally well-adapted to the most extreme environment in polar and alpine regions where harsh conditions on aridity, radiation exposure, and scarce water availability reduce the number of competitors. Indeed, the estimated origin of neuropogonoid lichens in the late Miocene (Figure 2b, Table 3) coincides with a time when the two centers of diversity, such as alpine zones in South America and subantarctic regions in the southern hemisphere (e.g., northern Andes, southern Chile, New Zealand) were highly impacted by (1) tectonic movements (e.g., maximum height of Andes) and subsequent proliferation of arid exposed cold deserts and (2) by glaciation of west Antarctica after the set of circumpolar current and subsequent triggering of tundra habitats [106,107]. Ultimately, the burst of diversification of neuropogonoid lichens took place around the Pliocene-Pleistocene boundary (~3 myr) or early Pleistocene (~1.5 myr) (Figure 2b, Table 3). In particular, our results shed light on the role of New Zealand in neuropogonoid lichen diversification bringing new insights to the biogeography of this group. Early colonization and diversification in New Zealand were inferred in the Miocene-Pliocene boundary (4.91–8.53 myr) which led to an ancient Amphipacific-disjunction at species level as well as more recent Pleistocene colonization events by sorediate species (Figure 1 and Figure 2). Long-distance dispersal (LDD) is the most plausible hypotheses for both ancient and recent colonization events since they postdate the split of the major landmasses. Indeed, this amphi-pacific pattern mediated by LDD is being widely observed in the lichen flora of New Zealand [108] where the climate became rapidly cool-temperate since the late Miocene and Pliocene being more similar to that of central Chile by that time [109].

## 5. Conclusions

The reference-based RAD sequencing method has resulted in strongly supported phylogenetic hypotheses of neuropogonoid lichens showing its power to resolve phylogenetic relationships in the genus *Usnea*, which is considered one of the most complicated groups of lichenized fungi. Large-scale genomic data in combination with a first-time full taxon coverage has led to: (1) the reconstruction of the backbone phylogeny for neuropogonoid lichens, (2) the inference of the evolutionary timeframe, and (3) the contribution to our knowledge of species diversity in the group. The massive genomic data obtained, coupled with morphological and chemical examinations, resolved two species complexes (*U. perpusilla* complex, *U. trachycarpa* complex) and circumscribed species endemic to New Zealand and the Kerguelen, respectively. Two species are newly described here, and three species names have been resurrected. Increasing aridity and global cooling during the late Miocene and the Pliocene-Pleistocene boundary are suggested as major drivers of diversification in neuropogonoid lichens. While this study provided a robust phylogeny of neuropogonoid lichens, it raises new questions about the extrinsic (e.g., historical contingency) and intrinsic factors (e.g., reproductive strategy) that may have interplayed to shape the genomic diversity and distributional ranges within and across both hemispheres of earth.

## Figures and Tables

**Table 1:** Chemosyndromes identified following [65] for each species of the four clades of taxonomical interest (Clades A-D; Figure 1). + indicates chemosyndrome constantly or frequently present in high concentration, ± indicates chemosyndrome either sometimes present in high concentrations or present in low concentration.

Phylogenetic Clade	Species	Hypostrepsilic Acid	Fumarprotocetraric Acid	Psoromic Acid	Salazinic Acid	Neuropogolic Acid
A	*U. ciliata*		±	±	+	
A	*U. subcapillaris*		±	±	+	
A	*Undescribed taxon*			+	+	
A	*U. pseudocapillaris*			±	+	
B	*U. rohmederi*					
C	*U. perpusilla*			±		
D	*U. aymondiana*		±	±		
D	*U. fibriloides*				+	
D	*U. sphacelata*			±		
D	*U. subantarctica*				+	
D	*U. trachycarpa*	±		±	±	±
D	*U. trachycarpoides*				+	
D	*U. hyyppae*				±	

**Table 2:** Main summary statistics yielded by the three datasets generated by ipyrad from the three values of minimum taxon coverage tested. The three values aimed to represent (1) the default value, m = 4; (2) 25% of the total samples, m = 32; and (3) 50% of the total samples, m = 65, denoted as m4, m25, and m50, respectively. The main statistics provided are (1) number of loci filtered, (2) mean loci per sample, (3) standard deviation (SD) of number of loci per sample, (4) minimum number of loci per sample, (5) maximum number of loci per sample, (6) number of single nucleotide polymorphisms (N snps), and (6) percentage of missing data in the loci matrix.

Dataset	N Samples	N Loci Filtered	Mean Loci per Sample	SD Loci per Sample	Min Loci	Max Loci	N snps	% Missing Data
m4	126	21,831	5917.376	3619.83	938	15,073	381,659	82.91
m25	126	8698	4705.896	2340.84	804	8182	271,485	76.22
m50	126	4504	3180.304	1141.65	706	4442	166,242	72.411

**Table 3:** Node ages (myr) and Bootstrapped variance (BV) intervals inferred through TreePL for Neuropogonoid species.

Species	Node Age	BV
*U. acromelana*	5.56	3.64–6.89
*U. antarctica*	1.8	0.49–3.09
*U. aurantiacoatra*	1.8	0.49–3.09
*U. aymondiana*	6.13	5.31–7.23
*U. ciliata*	2.75	1.14–5.91
*U. fibriloides*	5.09	3.62–6.58
*U. hyyppae*	1.36	0.43–2.27
*U. lambii*	5.75	2.66–7.72
*U. messutiae*	2.60	1–4.93
*U. pallidocarpa*	2.60	1–4.93
*U. patagonica*	7.3	4.91–8.54
*U. perpusilla*	5.45	2.79–7.34
*U. pseudocapillaris*	2.42	0.88–5.22
*U. rohmederi*	2.68	1.19–5.81
*U. sphacelata*	2.01	0.95–3.77
*U. subantarctica*	2.75	0.95–4.25
*U. subcapillaris*	2.75	1.14–5.91
*U. taylorii*	3.51	1.69–5.58
*U. trachycarpa*	2.01	0.95–3.77
*U. trachycarpoides*	1.36	0.43–2.27
*U. ushuaiensis*	2.68	1.19–5.81

## Data Availability

The data that support the findings of this study are openly available in the Short Read Archive (SRA), through the BioProject accession PRJNA902355 (https://www.ncbi.nlm.nih.gov/bioproject/PRJNA902355 (accessed on 7 January 2023)). Accession numbers for RADseq raw sequences are listed in Table S1.

## Supplementary Materials

The following supporting information can be downloaded at: https://www.mdpi.com/article/10.3390/jof9010099/s1, Figure S1: (a) RAxML-NG trees for m4, m25, and m50 settings of minimum taxon coverage. (b) RAxML trees for m4, m25, and m50 settings of minimum taxon coverage. Bootstrap support values (BS) are indicated for all nodes.; Figure S2: SVDquartets species tree of neuropogonoid lichens for m25 and m50 datasets; Figure S3: RAxML trees of the reduced dataset (i.e., one specimen per species) for m4, m25 and m50 settings; Figure S4: Tree topology and BABA-ABBA scheme used for *D*-stat tests performed in ipyrad. Table S1: Data information and NCBI SRA accessions of all individuals sampled; Table S2: Morphological characterization of the species included in the phylogenetic clades of taxonomic interest; Table S3: Number of loci obtained per sample for the three datasets (i.e., m4, m25, and m50) tested for the present study and number of raw reads obtained after demultiplexing step.; Table S4: Chemical substances obtained through HPTLC analysis and chemotypes assigned for the species of taxonomic interests (clades A-D). Table S5: Summary of *D*-statistic analysis performed for all tests resulted from all possible sample combinations in a four-taxon tree [(((P1,P2),P3),P4)].
