# Supplementary material for: Reference-Based RADseq Unravels the Evolutionary History of Polar Species in ‘the Crux Lichenologorum’ Genus Usnea (Parmeliaceae, Ascomycota)
Source: J Fungi (Basel). 2023 Jan 11;9(1):99. doi: 10.3390/jof9010099 (PMC9865703; doi:10.3390/jof9010099)
Supplement: Supplementary file 1 [file jof-09-00099-s001.zip › jof-2113956-SI.docx]

**Figure S1:** (a) RAxML-NG trees for m4, m25 and m50 settings of minimum taxon coverage. (b) RAxML trees for m4, m25 and m50 settings of minimum taxon coverage. Bootstrap support values (BS) are indicated for all nodes.

**(a)**


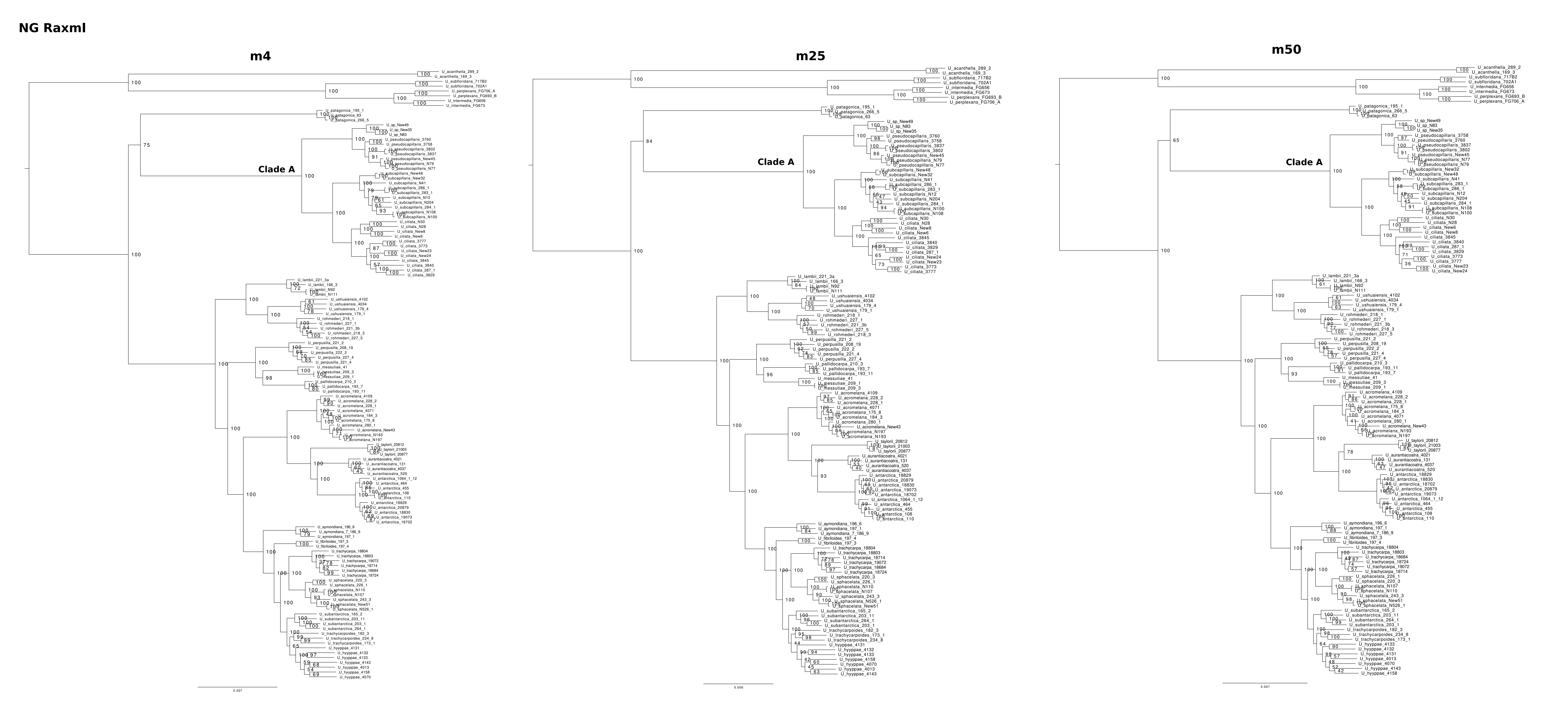


**Fig. S1 (cont.)**

**
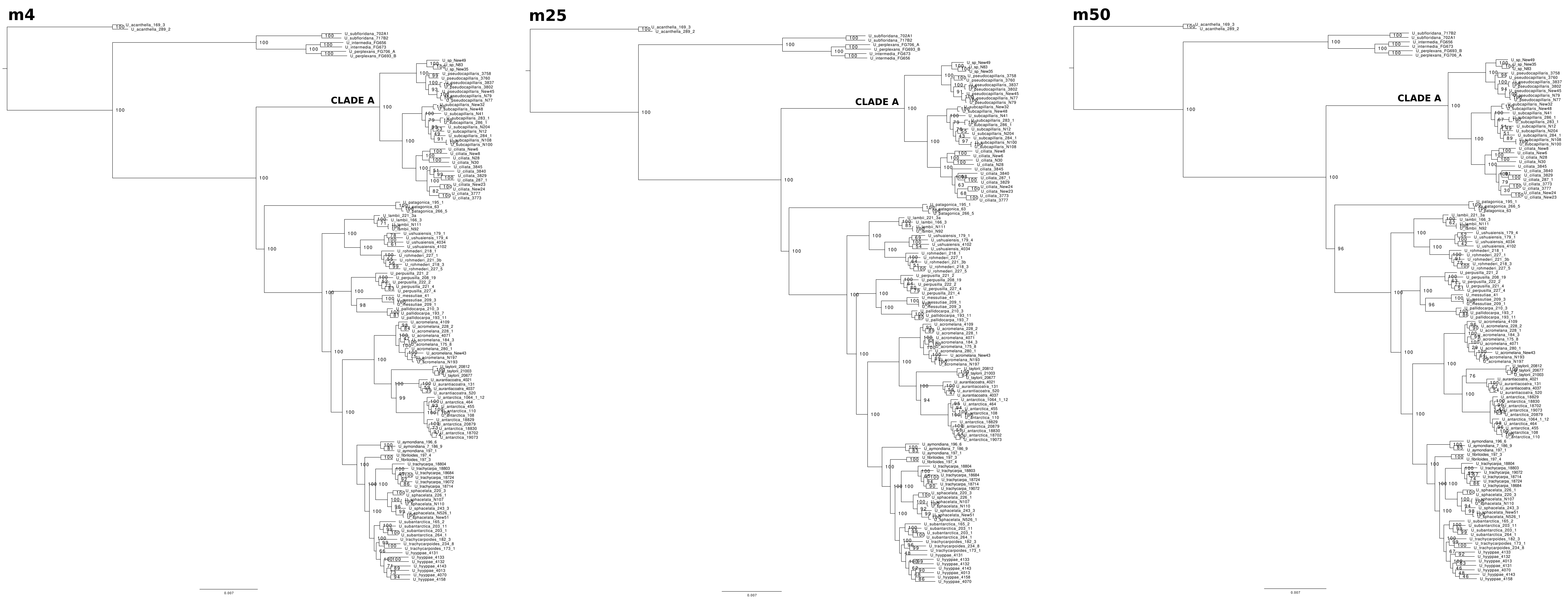
**

**(b)**

**Figure S2:** SVDquartets species tree of neuropogonoid lichens for m25 and m50 dataset. Bootstrap Supports (BS) are indicated for each node.


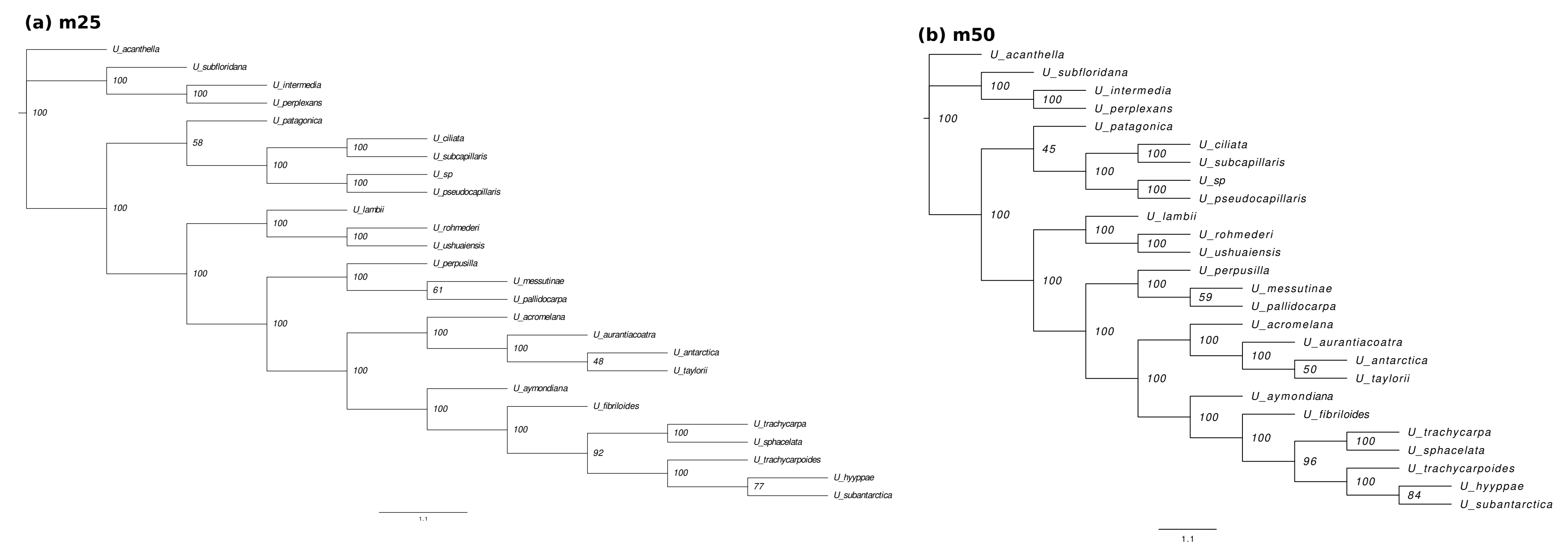


**Figure S3**: RAxML trees of for the reduced dataset (i.e. one specimen per species) of m4, m25 and m50. Bootstrap support values (BS) are indicated for all nodes.


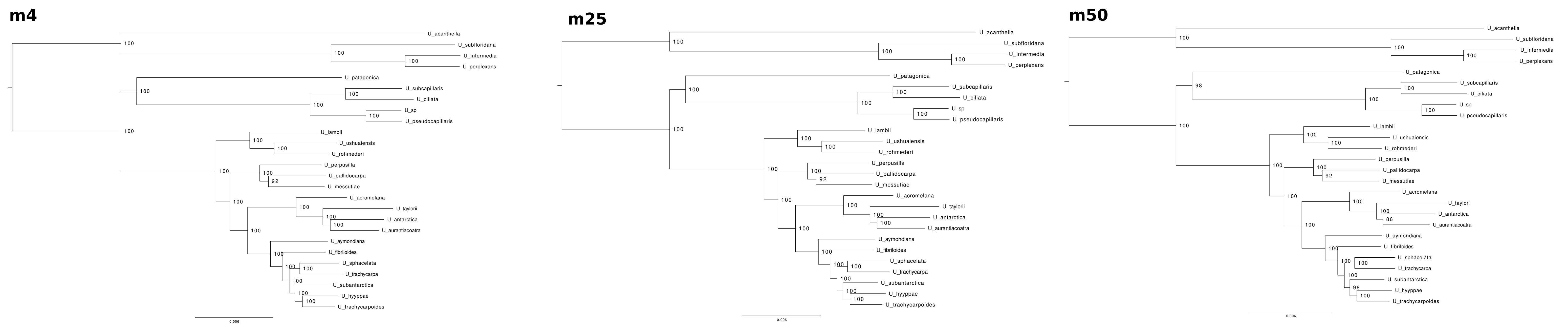


**Figure S4:** Tree topology and BABA-ABBA scheme used for D-stat test performed in Ipyrad.


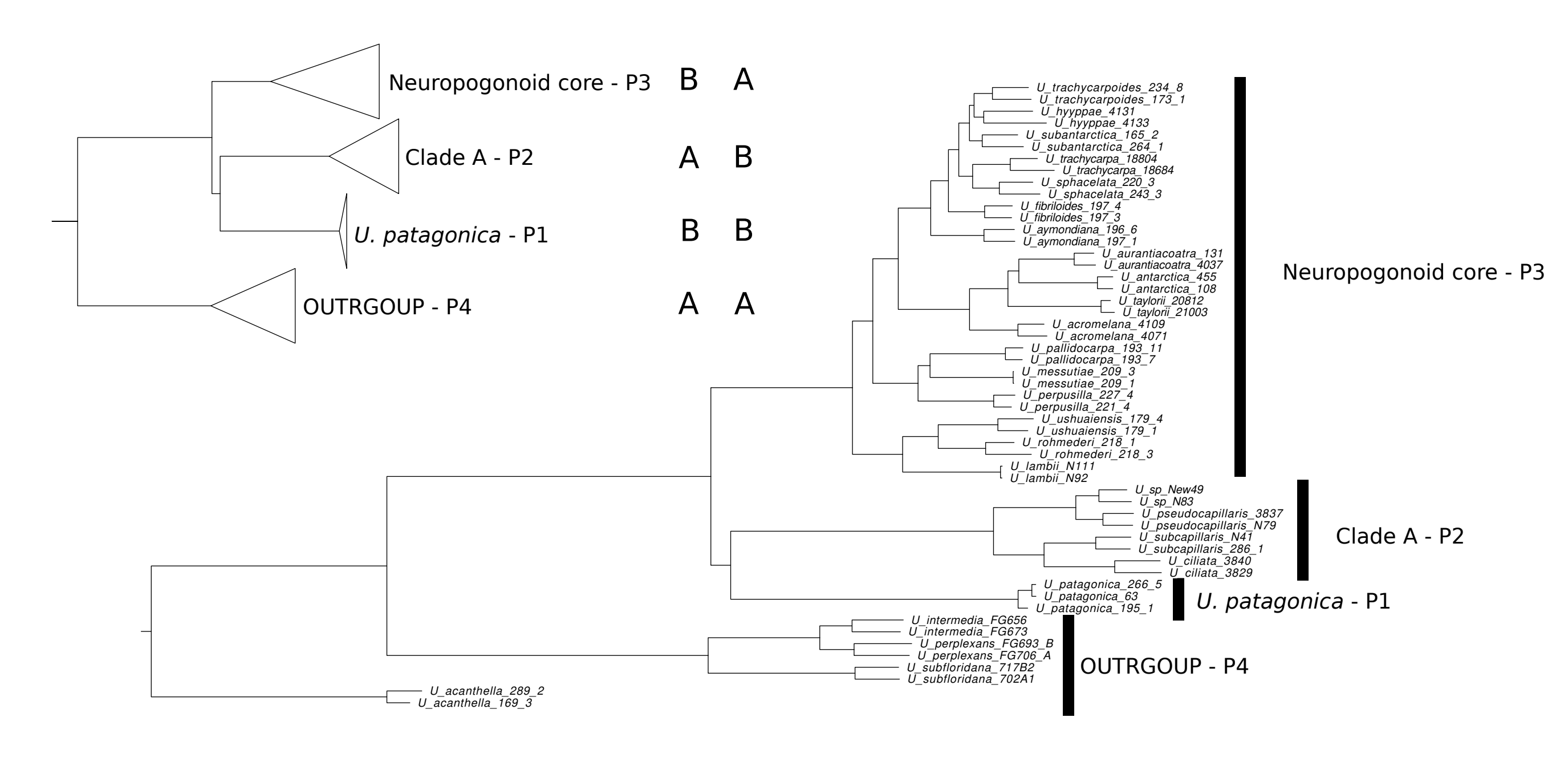


**Table S1.** Data information and NCBI SRA accessions of all individuals sampled. Species names newly described in the present study are marked in bold.

| **Species** | **Voucher** | **Collector** | **Herbarium code** | **Locality** | **NCBI SRA Accession** |
| --- | --- | --- | --- | --- | --- |
| *Usnea acanthella* (I.M. Lamb) F.J. Walker | 169_3 | Z. Palice s.n. 6 Mar 2003 (F) | F | Ecuador, Carchi | SAMN31757397 |
| *Usnea acanthella* (I.M. Lamb) F.J. Walker | 289_2 | N. Wirtz, H. T. Lumbsch 19320c & A. Ramirez (F) | F | Peru, Parque Nacional, Huascaran | SAMN31757398 |
| *Usnea acromelana* Stirt. | 175_8 | H.T. Lumbsch 2002 | F | Chile, Tierra del Fuego, Glaciar Martial | SAMN31757399 |
| *Usnea acromelana* Stirt. | 184_3 | N. Wirtz & M.I. Messuti 12/2003 | F | Chile, Tierra del Fuego, Glaciar Martial, 710m | SAMN31757400 |
| *Usnea acromelana* Stirt. | 228_1 | N. Wirtz & M.I. Messuti 12/2003 | F | Chile, Tierra del Fuego, Lapataia Bay, 55m | SAMN31757401 |
| *Usnea acromelana* Stirt. | 228_2 | N. Wirtz & M.I. Messuti 12/2003 | F | Chile, Tierra del Fuego, Lapataia Bay, 55m | SAMN31757402 |
| *Usnea acromelana* Stirt. | 280_1 | A. Knight 04/2003 | F | New Zealand, Otago, Pisa Range, 1700m | SAMN31757403 |
| *Usnea acromelana* Stirt. | 4071 | F.T. Grewe, Todd Widhelm, M. von Konrat, J. Larrain, 2017 | F | Chile, Navarino Island | SAMN31757404 |
| *Usnea acromelana* Stirt. | 4109 | F. Grewe, T. Widhelm, M. von Konrat, J. Larrain, 2017 | F | Chile, Navarino Island | SAMN31757405 |
| *Usnea acromelana* Stirt. | N193 | C. Printzen, FR-0265513 | FR | New Zealand, Otago, Sir William Peak, Mt. Earnslaw | SAMN31757406 |
| *Usnea acromelana* Stirt. | N197 | C. Printzen, FR-0265513 | FR | New Zealand, Otago, Sir William Peak, Mt. Earnslaw | SAMN31757407 |
| *Usnea antarctica* Du Rietz | 1064_1_12 | C. Prinzen 1064-1 | F | Antarctica, Livingston Island, Punta Hespérides | SAMN31757408 |
| *Usnea antarctica* Du Rietz | 108 | F. Grewe | F | Antarctica, Livingston Island, Caleta Argentina | SAMN31757409 |
| *Usnea antarctica* Du Rietz | 110 | F. Grewe | F | Antarctica, Livingston Island, Caleta Argentina | SAMN31757410 |
| *Usnea antarctica* Du Rietz | 18702 | D. Ertz | F | Kerguelen Islands | SAMN31757411 |
| *Usnea antarctica* Du Rietz | 18829 | D. Ertz | F | Kerguelen Islands | SAMN31757412 |
| *Usnea antarctica* Du Rietz | 18830 | D. Ertz | F | Kerguelen Islands | SAMN31757413 |
| *Usnea antarctica* Du Rietz | 19073 | D. Ertz | F | Kerguelen Islands | SAMN31757414 |
| *Usnea antarctica* Du Rietz | 20879 | D. Ertz | F | Kerguelen Islands | SAMN31757415 |
| *Usnea antarctica* Du Rietz | 455 | F. Grewe | F | Antarctica, Hannah Point | SAMN31757416 |
| *Usnea antarctica* Du Rietz | 464 | F. Grewe | F | Antarctica, Hannah Point | SAMN31757417 |
| *Usnea aurantiacoatra* (Jacq.) Bory | 4021 | F. Grewe, T. Widhelm, M. von Konrat, J. Larrain, 2017 | F | Chile, Navarino Island | SAMN31757418 |
| *Usnea aurantiacoatra* (Jacq.) Bory | 4037 | F. Grewe, T. Widhelm, M. von Konrat, J. Larrain, 2017 | F | Chile, Navarino Island | SAMN31757419 |
| *Usnea aurantiacoatra* (Jacq.) Bory | 131 | F. Grewe | F | Antarctica, Livingston Island, Caleta Argentina | SAMN31757420 |
| *Usnea aurantiacoatra* (Jacq.) Bory | 520 | F. Grewe | F | Antarctica, Livingston Island, Nunnatak | SAMN31757421 |
| ***Usnea aymondiana* Otero, Bárcenas-Peña, Lumbsch & Grewe** | 7-186_9 | N. Wirtz, M.I. Messuti 12/2003 | F | Argentina, Santa Cruz Province, Monte Aymond | SAMN31757422 |
| ***Usnea aymondiana* Otero, Bárcenas-Peña, Lumbsch & Grewe** | 196_6 | N. Wirtz, M.I. Messuti 12/2003 | F | Argentina, Santa Cruz Province, Monte Aymond | SAMN31757423 |
| ***Usnea aymondiana* Otero, Bárcenas-Peña, Lumbsch & Grewe** | 197_1 | N. Wirtz, M.I. Messuti 12/2003 | F | Argentina, Santa Cruz Province, Monte Aymond | SAMN31757424 |
| *Usnea ciliata* (Nyl.) Du Rietz | 287_1 | K. Spencer 11/2004 | F | New Zealand, Otago, Old Man Range | SAMN31757425 |
| *Usnea ciliata* (Nyl.) Du Rietz | 3773 | F. Grewe, T. Widhelm | F | New Zealand, Otago, Pisa Range | SAMN31757426 |
| *Usnea ciliata* (Nyl.) Du Rietz | 3777 | F. Grewe, T. Widhelm | F | New Zealand, Otago, Pisa Range | SAMN31757427 |
| *Usnea ciliata* (Nyl.) Du Rietz | 3829 | F. Grewe, T. Widhelm | F | New Zealand, Otago, Old Man Range | SAMN31757428 |
| *Usnea ciliata* (Nyl.) Du Rietz | 3840 | F. Grewe, T. Widhelm | F | New Zealand, Otago, Old Man Range | SAMN31757429 |
| *Usnea ciliata* (Nyl.) Du Rietz | 3845 | F. Grewe, T. Widhelm | F | New Zealand, Otago, Old Man Range | SAMN31757430 |
| *Usnea ciliata* (Nyl.) Du Rietz | N27 | C. Printzen FR-0265519 | FR (antiguo N28) | New Zealand, Canterbury, Cragieburn Range | SAMN31757431 |
| *Usnea ciliata* (Nyl.) Du Rietz | N29 | C. Printzen FR-0265519 | FR (antiguo N30) | New Zealand, Canterbury, Cragieburn Range | SAMN31757432 |
| *Usnea ciliata* (Nyl.) Du Rietz | New23 | C. Printzen FR-0265504 | FR | New Zealand, Canterbury, Cragieburn Range | SAMN31757433 |
| *Usnea ciliata* (Nyl.) Du Rietz | New24 | C. Printzen FR-0265504 | FR | New Zealand, Canterbury, Cragieburn Range | SAMN31757434 |
| *Usnea ciliata* (Nyl.) Du Rietz | New6 | C. Printzen FR-0265503 | FR | New Zealand, Canterbury, Cragieburn Range | SAMN31757435 |
| *Usnea ciliata* (Nyl.) Du Rietz | New8 | C. Printzen FR-0265516 | FR | New Zealand, Canterbury, Cragieburn Range | SAMN31757436 |
| ***Usnea fibriloides* Otero, Bárcenas-Peña, Lumbsch & Grewe** | 197_3 | N. Wirtz, M.I. Messuti 12/2003 | F | Argentina, Santa Cruz Province, Monte Aymond | SAMN31757437 |
| ***Usnea fibriloides* Otero, Bárcenas-Peña, Lumbsch & Grewe** | 197_4 | N. Wirtz, M.I. Messuti 12/2003 | F | Argentina, Santa Cruz Province, Monte Aymond | SAMN31757438 |
| *Usnea hyyppae* Räsänen | 4013 | F. Grewe, T. Widhelm, M. von Konrat, J. Larrain, 2017 | F | Chile, Navarino Island | SAMN31757439 |
| *Usnea hyyppae* Räsänen | 4070 | F. Grewe, T. Widhelm, M. von Konrat, J. Larrain, 2017 | F | Chile, Navarino Island | SAMN31757440 |
| *Usnea hyyppae* Räsänen | 4131 | F. Grewe, T. Widhelm, M. von Konrat, J. Larrain, 2017 | F | Chile, Navarino Island | SAMN31757441 |
| *Usnea hyyppae* Räsänen | 4132 | F. Grewe, T. Widhelm, M. von Konrat, J. Larrain, 2017 | F | Chile, Navarino Island | SAMN31757442 |
| *Usnea hyyppae* Räsänen | 4133 | F. Grewe, T. Widhelm, M. von Konrat, J. Larrain, 2017 | F | Chile, Navarino Island | SAMN31757443 |
| *Usnea hyyppae* Räsänen | 4143 | F. Grewe, T. Widhelm, M. von Konrat, J. Larrain, 2017 | F | Chile, Navarino Island | SAMN31757444 |
| *Usnea hyyppae* Räsänen | 4158 | F. Grewe, T. Widhelm, M. von Konrat, J. Larrain, 2017 | F | Chile, Navarino Island | SAMN31757445 |
| *Usnea sp.* | N83 | C. Printzen FR-0265509 | FR | New Zealand, Otago, Pisa Range | SAMN31757446 |
| *Usnea sp.* | New35 | C. Printzen FR-0265509 | FR | New Zealand, Otago, Pisa Range | SAMN31757447 |
| *Usnea sp.* | New49 | C. Printzen FR-0265509 | FR | New Zealand, Otago, Pisa Range | SAMN31757448 |
| *Usnea intermedia* (A. Massal.) Jatta | FG656 | H.T. Lumbsch, F. Grewe | F | Germany, Baden-Württemberg, Schwarzwald, Todtmoos | SAMN31757449 |
| *Usnea intermedia* (A. Massal.) Jatta | FG673 | H.T. Lumbsch, F. Grewe | F | Austria, Salzburg | SAMN31757450 |
| *Usnea lambii* (Imshaug) Wirtz & Lumbsch | 166_3 | L. Sancho, A. Green 01/2003 | F | Antarctica, Victoria Land, Nunatak | SAMN31757451 |
| *Usnea lambii* (Imshaug) Wirtz & Lumbsch | 221_3a | N. Wirtz, M.I. Messuti 12/2003 | F | Argentina, Rio Negro, Bariloche, C. Catedral | SAMN31757452 |
| *Usnea lambii* (Imshaug) Wirtz & Lumbsch | N111 | C. Printzen FR-0265514 | FR | New Zealand, Otago, Sir William Peak, Mt. Earnslaw | SAMN31757453 |
| *Usnea lambii* (Imshaug) Wirtz & Lumbsch | N92 | C. Printzen FR-0265513 | FR | New Zealand, Otago, Sir William Peak, Mt. Earnslaw | SAMN31757454 |
| *Usnea messutiae* Wirtz & Lumbsch | 209_1 | N. Wirtz & M.I. Messuti 12/2003 | F | Argentina, Rio Negro, C. Catedral, 1850m | SAMN31757455 |
| *Usnea messutiae* Wirtz & Lumbsch | 209_3 | N. Wirtz & M.I. Messuti 12/2003 | F | Argentina, Rio Negro, C. Catedral, 1850m | SAMN31757456 |
| *Usnea messutiae* Wirtz & Lumbsch | 41 | Z. Palice, Soldán 2000 | F | Ecuador, Chimborazo, 4600m | SAMN31757457 |
| *Usnea pallidocarpa* Wirtz & Lumbsch | 193_11 | N. Wirtz & M.I. Messuti 12/2003 | F | Argentina, Rio Negro, C. Catedral, 1900m | SAMN31757458 |
| *Usnea pallidocarpa* Wirtz & Lumbsch | 193_7 | N. Wirtz & M.I. Messuti 12/2003 | F | Argentina, Rio Negro, C. Catedral, 1900m | SAMN31757459 |
| *Usnea pallidocarpa* Wirtz & Lumbsch | 210_3 | N. Wirtz & M.I. Messuti 12/2003 | F | Argentina, Rio Negro, C. Catedral, 1850m | SAMN31757460 |
| *Usnea patagonica* F.J. Walker | 195_1 | N. Wirtz, M.I. Messuti 12/2003 | F | Argentina, Santa Cruz Province, Monte Aymond | SAMN31757461 |
| *Usnea patagonica* F.J. Walker | 266_5 | N. Wirtz, H.T. Lumbsch & A. Ramírez 11/2004 | F | Peru, Ancash, Nevado Pastoruri, 4820m | SAMN31757462 |
| *Usnea patagonica* F.J. Walker | 63 | Z. Palice 1999 | F | Ecuador, Chimborazo | SAMN31757463 |
| *Usnea perplexans* Stirt. | FG693_B | H.T. Lumbsch, F. Grewe | F | Austria, Tyrol, Schranzberg | SAMN31757464 |
| *Usnea perplexans* Stirt. | FG706_A | H.T. Lumbsch, F. Grewe | F | Austria, Tyrol, Gleins | SAMN31757465 |
| *Usnea perpusilla* (I.M. Lamb) F.J. Walker | 208_19 | N. Wirtz & M.I. Messuti 12/2003 | F | Argentina, Rio Negro, C. Catedral, 1850m | SAMN31757466 |
| *Usnea perpusilla* (I.M. Lamb) F.J. Walker | 221_2 | N. Wirtz & M.I. Messuti 12/2003 | F | Argentina, Rio Negro, C. Catedral 2200m | SAMN31757467 |
| *Usnea perpusilla* (I.M. Lamb) F.J. Walker | 221_4 | N. Wirtz & M.I. Messuti 12/2003 | F | Argentina, Rio Negro, C. Catedral 2200m | SAMN31757468 |
| *Usnea perpusilla* (I.M. Lamb) F.J. Walker | 222_2 | N. Wirtz & M.I. Messuti 12/2003 | F | Argentina, Rio Negro, C. Catedral 2200m | SAMN31757469 |
| *Usnea perpusilla* (I.M. Lamb) F.J. Walker | 227_4 | N. Wirtz & M.I. Messuti 12/2003 | F | Argentina, Rio Negro, C. Catedral 2200m | SAMN31757470 |
| *Usnea pseudocapillaris* F.J. Walker | 3758 | F. Grewe, T. Widhelm | F | New Zealand, Otago, Pisa Range | SAMN31757471 |
| *Usnea pseudocapillaris* F.J. Walker | 3760 | F. Grewe, T. Widhelm | F | New Zealand, Otago, Pisa Range | SAMN31757472 |
| *Usnea pseudocapillaris* F.J. Walker | 3802 | F. Grewe, T. Widhelm | F | New Zealand, Otago, Old Man Range | SAMN31757473 |
| *Usnea pseudocapillaris* F.J. Walker | 3837 | F. Grewe, T. Widhelm | F | New Zealand, Otago, Old Man Range | SAMN31757474 |
| *Usnea pseudocapillaris* F.J. Walker | N77 | C. Printzen FR-0265529 | FR | New Zealand, Otago, Pisa Range | SAMN31757475 |
| *Usnea pseudocapillaris* F.J. Walker | N79 | C. Printzen FR-0265529 | FR | New Zealand, Otago, Pisa Range | SAMN31757476 |
| *Usnea pseudocapillaris* F.J. Walker | New45 | C. Printzen FR-0265529 | FR confirm! | New Zealand, Otago, Pisa Range | SAMN31757477 |
| *Usnea rohmederi* (I.M. Lamb) I.M. Lamb | 218_1 | N. Wirtz & M.I. Messuti 12/2003 | F | Argentina, Rio Negro, C. Catedral 2200m | SAMN31757478 |
| *Usnea rohmederi* (I.M. Lamb) I.M. Lamb | 218_3 | N. Wirtz & M.I. Messuti 12/2003 | F | Argentina, Rio Negro, C. Catedral 2200m | SAMN31757479 |
| *Usnea rohmederi* (I.M. Lamb) I.M. Lamb | 221_3b | N. Wirtz & M.I. Messuti 12/2003 | F | Argentina, Rio Negro, C. Catedral 2200m | SAMN31757480 |
| *Usnea rohmederi* (I.M. Lamb) I.M. Lamb | 227_1 | N. Wirtz & M.I. Messuti 12/2003 | F | Argentina, Rio Negro, C. Catedral 2200m | SAMN31757481 |
| *Usnea rohmederi* (I.M. Lamb) I.M. Lamb | 227_5 | N. Wirtz & M.I. Messuti 12/2003 | F | Argentina, Rio Negro, C. Catedral 2200m | SAMN31757482 |
| *Usnea trachycarpa* (Stirt.) Müll. Arg | 18684 | D. Ertz | F | Kerguelen Islands | SAMN31757483 |
| *Usnea trachycarpa* (Stirt.) Müll. Arg | 18714 | D. Ertz | F | Kerguelen Islands | SAMN31757484 |
| *Usnea trachycarpa* (Stirt.) Müll. Arg | 18724 | D. Ertz | F | Kerguelen Islands | SAMN31757485 |
| *Usnea trachycarpa* (Stirt.) Müll. Arg | 18803 | D. Ertz | F | Kerguelen Islands | SAMN31757486 |
| *Usnea trachycarpa* (Stirt.) Müll. Arg | 18804 | D. Ertz | F | Kerguelen Islands | SAMN31757487 |
| *Usnea trachycarpa* (Stirt.) Müll. Arg | 19072 | D. Ertz | F | Kerguelen Islands | SAMN31757488 |
| *Usnea sphacelata* R. Br. | 220_3 | N. Wirtz & M.I. Messuti 12/2003 | F | Argentina, Rio Negro, C. Catedral 2200m | SAMN31757489 |
| *Usnea sphacelata* R. Br. | 226_1 | N. Wirtz & M.I. Messuti 12/2003 | F | Argentina, Rio Negro, C. Catedral 2200m | SAMN31757490 |
| *Usnea sphacelata* R. Br. | 243_3 | A.V. Larsen 07/1996 | F | Greenland, Disko | SAMN31757491 |
| *Usnea sphacelata* R. Br. | N107 | C. Printzen FR-0265515 | FR | New Zealand, Otago, Sir William Peak, Mt. Earnslaw | SAMN31757492 |
| *Usnea sphacelata* R. Br. | N110 | C. Printzen FR-0265514 | FR | New Zealand, Otago, Sir William Peak, Mt. Earnslaw | SAMN31757493 |
| *Usnea sphacelata* R. Br. | N526_1 | C. Printzen FR-0265511 | FR | New Zealand, Otago, Sir William Peak, Mt. Earnslaw | SAMN31757494 |
| *Usnea sphacelata* R. Br. | New43 | C. Printzen FR-0265511 | FR | New Zealand, Otago, Sir William Peak, Mt. Earnslaw | SAMN31757495 |
| *Usnea sphacelata* R. Br. | New51 | C. Printzen FR-0265511 | FR | New Zealand, Otago, Sir William Peak, Mt. Earnslaw | SAMN31757496 |
| *Usnea subantarctica* F.J. Walker | 165_2 | S. Ott 1999 | F | Antarctica, Lagoon Island | SAMN31757497 |
| *Usnea subantarctica* F.J. Walker | 203_1 | N. Wirtz & M.I. Messuti 12/2003 | F | Argentina, Santa Cruz, El Chalten | SAMN31757498 |
| *Usnea subantarctica* F.J. Walker | 203_11 | N. Wirtz & M.I. Messuti 12/2003 | F | Argentina, Santa Cruz, El Chalten | SAMN31757499 |
| *Usnea subantarctica* F.J. Walker | 264_1 | N. Wirtz & M.I. Messuti 12/2003 | F | Chile, Tierra del Fuego, Glacier Martial | SAMN31757500 |
| *Usnea subcapillaris* (D.J. Galloway) F.J. Walker | 283_1 | D. J. Galloway 53636 (OTA) | F | New Zealand, Otago | SAMN31757501 |
| *Usnea subcapillaris* (D.J. Galloway) F.J. Walker | 284_1 | D.J. Galloway 02/1998 | F | New Zealand, Otago, Lammerlaw Range | SAMN31757502 |
| *Usnea subcapillaris* (D.J. Galloway) F.J. Walker | 286_1 | K. Spencer 58829 (OTA) | F | New Zealand, Otago | SAMN31757503 |
| *Usnea subcapillaris* (D.J. Galloway) F.J. Walker | N100 | C. Printzen FR-0265510 | FR | New Zealand, Otago, Sir William Peak, Mt. Earnslaw | SAMN31757504 |
| *Usnea subcapillaris* (D.J. Galloway) F.J. Walker | N108 | C. Printzen FR-0265514 | FR | New Zealand, Otago, Sir William Peak, Mt. Earnslaw | SAMN31757505 |
| *Usnea subcapillaris* (D.J. Galloway) F.J. Walker | N12 | C. Printzen FR-0265517 | FR | New Zealand, Canterbury, Cragieburn Range | SAMN31757506 |
| *Usnea subcapillaris* (D.J. Galloway) F.J. Walker | N204 | C. Printzen FR-0265523 | FR | New Zealand, Otago, Old Man Range | SAMN31757507 |
| *Usnea subcapillaris* (D.J. Galloway) F.J. Walker | N41 | C. Printzen FR-0265520 | FR | New Zealand, Otago, Old Man Range | SAMN31757508 |
| *Usnea subcapillaris* (D.J. Galloway) F.J. Walker | New32 | C. Printzen FR-0265508 | FR | New Zealand, Otago, Pisa Range | SAMN31757509 |
| *Usnea subcapillaris* (D.J. Galloway) F.J. Walker | New48 | C. Printzen FR-0265508 | FR | New Zealand, Otago, Pisa Range | SAMN31757510 |
| *Usnea subfloridana* Stirt. | 702A1 | H.T. Lumbsch, F. Grewe | F | Austria, Tyrol, Gleins | SAMN31757511 |
| *Usnea subfloridana* Stirt. | 717B2 | H.T. Lumbsch, F. Grewe | F | Austris, Tyrol, Froneben | SAMN31757512 |
| *Usnea taylorii* Hook. f. & Taylor | 20812 | D. Ertz | F | Kerguelen Islands | SAMN31757513 |
| *Usnea taylorii* Hook. f. & Taylor | 21003 | D. Ertz | F | Kerguelen Islands | SAMN31757514 |
| *Usnea taylorii* Hook. f. & Taylor | 20877 | D. Ertz | F | Kerguelen Islands | SAMN31757515 |
| *Usnea trachycarpoides* (Vain.) C.W. Dodge | 173_1 | H.T. Lumbsch 2002 |  | Chile, Tierra del Fuego, Lapataia Bay | SAMN31757516 |
| *Usnea trachycarpoides* (Vain.) C.W. Dodge | 182_3 | N. Wirtz & M.I. Messuti 12/2003 | F | Chile, Tierra del Fuego, Glaciar Martial | SAMN31757517 |
| *Usnea trachycarpoides* (Vain.) C.W. Dodge | 234_8 | N. Wirtz & M.I. Messuti 12/2003 | F | Chile, Tierra del Fuego, Lapataia Bay | SAMN31757518 |
| *Usnea ushuaiensis* (I.M. Lamb) Wirtz, Printzen & Lumbsch | 179_1 | N. Wirtz & M.I. Messuti 12/2003 | F | Chile, Tierra del Fuego, Glaciar Martial | SAMN31757519 |
| *Usnea ushuaiensis* (I.M. Lamb) Wirtz, Printzen & Lumbsch | 179_4 | N. Wirtz & M.I. Messuti 12/2003 | F | Chile, Tierra del Fuego, Glaciar Martial | SAMN31757520 |
| *Usnea ushuaiensis* (I.M. Lamb) Wirtz, Printzen & Lumbsch | 4034 | F. Grewe, T. Widhelm, M. von Konrat, J. Larrain, 2017 | F | Chile, Navarino Island | SAMN31757521 |
| *Usnea ushuaiensis* (I.M. Lamb) Wirtz, Printzen & Lumbsch | 4102 | F. Grewe, T. Widhelm, M. von Konrat, J. Larrain, 2017 | F | Chile, Navarino Island | SAMN31757522 |

**Table S2.** Morphological characterization of the species included in the phylogenetic clades of taxonomic interest.

| **Phylogenetic Clade** | **Species** | **Habit** | **Holdfast** | **Branching** | **Pigmentation pattern** | **Surface ornamentation** | **Papillae** | **Fibrils** | **Internal structure** | **Apothecia** | **Vegetative propagules** |
| --- | --- | --- | --- | --- | --- | --- | --- | --- | --- | --- | --- |
| ***A*** | ***U. ciliata*** | Erect | Proliferating | Moderate | Yellowish to black-violaceaous towards the apices | Smooth, black-edge annulations | no | no | Cortex thick, medulla compact, axis compact less than half | terminal/subterminal, black disc, few black short excipular rays | Plane rounded soredia, with black spots* |
| ***A*** | ***U. subcapillaris*** | Pendulous-subpendulous | Delimited | Extensive | Yellowish to black variegation, black apices | Smooth | no | no | Cortex thick, medulla compact, axis compact more than half | lateral, black disc, frequent black long excipular rays | no |
| ***A*** | ***Undescribed taxon*** | Erect | Proliferating | Rare | Yellowish, black only tips | Smooth, black-edge annulations | no | no | Cortex thin, medulla compact, axis compact more than half | terminal/subterminal, black disc, few black short excipular rays | no |
| ***A*** | ***U. pseudocapillaris*** | Erect-subpendulous | Delimited | Extensive | Yellowish to black towards the apices | Smooth | no | no | Cortex thin, medulla compact, axis compact more than half | no | Plane rounded to confluent or minute punctiform soredia with black spots |
| ***B*** | ***U. rohmederi*** | Erect | Delimited | Rich | Yellowish, black variegated at the apices | Smooth | no | absent to abundant | Cortex thick, medulla lax, axis compact half | lateral, black disc, frequent black long excipular rays, sometimes also variegated | no |
| ***C*** | ***U. perpusilla*** | Erect | Proliferating | Rich | Yellowish to black towards the apices rarely slightly variegated | Smooth | no | no | Cortex thick, medulla sublax, axis compact, variable width | lateral/subterminal, black disc, absent to spread black short excipular rays | no |
| ***D*** | ***U. aymondiana*** | Erect | Delimited | Moderate | Yellowish, black only tips | Rugose, abundant papillae and fibrils | yes | yes | Cortex thick, medulla sublax, axis compact less than half | subterminal, brownish-red disc, margin verrucose with fibrils, frequent long yellow excipular rays | no |
| ***D*** | ***U. fibriloides*** | Erect | Delimited | Rare | Yellowish, black only fibrils | Highly rugose, richly papillae, short and abundant fibrils | yes | yes | Cortex thick, medulla sublax, axis compact half | terminal, brownish-red disc, margin verrucose and abundant minute black fibrils | no |
| ***D*** | ***U. sphacelata*** | Erect | +- delimited / proliferating | Richly | Yellowish, black variegated at the apices | Smooth to spread papillae on main branches | yes | no | Cortex thin, medulla sublax, axis sublax half | no | Excavated/rounded to globose black pigmented |
| ***D*** | ***U. trachycarpa*** | Erect | Delimited | Moderate | Yellowish to black towards the apices | Richly black papillae and short thick fibrils | yes | yes | Cortex thick, medulla compact, axis compact more than half | subterminal, orange or orange and black discs, cupular/flattened, margin with short erect fibrils and papillae | no |
| ***D*** | ***U. subantarctica*** | Erect | Proliferating | Moderate | Yellowish, black variegated at the apices | Rugose, abundantly papillae, short spread fibrils | yes | yes | Cortex thick, medulla lax, axis compact half | no | Globose black pigmented |
| ***D*** | ***U. trachycarpoides*** | Erect | +- delimited / proliferating | Moderate | Yellowsih, black variegated at the apices | Rugose, absent to abundant papillae, long abundant fibrils | yes | yes | Cortex thick, medulla lax to sublax, axis compact half or more than half | terminal/subterminal, orange disc, margin with medium fibrils | no |
| ***D*** | ***U. hyyppae*** | Erect | +- delimited / proliferating | Variable | Yellowish/ black at the base, not/lightly variegated at the tips | Rugose, abundant papillae, long abundant fibrils | yes | yes | Cortex thick, medulla sublax axis compact half | terminal, orange disc, margin with very long / short thick fibrils | no |

**Table S3.** Number of loci obtained per sample for the three datasets (i.e. m4, m25, and m50) tested for the present study and number of raw reads obtained after demultiplexing step.

| **Sample** | **m4** | **m25** | **m50** | **Raw reads** |
| --- | --- | --- | --- | --- |
| U_acanthella_169_3 | 2615 | 2220 | 1838 | 1728449 |
| U_acanthella_289_2 | 938 | 804 | 706 | 1443120 |
| U_acromelana_175_8 | 1451 | 1391 | 1274 | 1960478 |
| U_acromelana_184_3 | 2169 | 2066 | 1873 | 1623670 |
| U_acromelana_228_1 | 4400 | 4151 | 3491 | 2056952 |
| U_acromelana_228_2 | 2741 | 2617 | 2332 | 1050276 |
| U_acromelana_280_1 | 1415 | 1340 | 1213 | 2008532 |
| U_acromelana_4071 | 11920 | 8007 | 4386 | 5918935 |
| U_acromelana_4109 | 9169 | 7565 | 4336 | 6366182 |
| U_acromelana_N193 | 2667 | 2482 | 2158 | 1004320 |
| U_acromelana_N197 | 3071 | 2825 | 2354 | 1076956 |
| U_antarctica_1064_1_12 | 14541 | 8147 | 4400 | 6180931 |
| U_antarctica_108 | 14713 | 8182 | 4411 | 6449276 |
| U_antarctica_110 | 11044 | 7877 | 4352 | 2402096 |
| U_antarctica_18702 | 10599 | 7659 | 4313 | 1591913 |
| U_antarctica_18829 | 10064 | 7622 | 4320 | 1772085 |
| U_antarctica_18830 | 8729 | 7086 | 4257 | 1837691 |
| U_antarctica_19073 | 10054 | 7656 | 4336 | 1778461 |
| U_antarctica_20879 | 11880 | 7843 | 4356 | 1736736 |
| U_antarctica_455 | 15073 | 8169 | 4406 | 6182543 |
| U_antarctica_464 | 8853 | 7373 | 4308 | 1942728 |
| U_aurantiacoatra_4021 | 9239 | 7475 | 4299 | 5704875 |
| U_aurantiacoatra_4037 | 11804 | 7894 | 4342 | 4358957 |
| U_aurantiacoatra_131 | 9908 | 7706 | 4332 | 6569624 |
| U_aurantiacoatra_520 | 8955 | 7502 | 4309 | 1489197 |
| U_aymondiana_7_186_9 | 1037 | 1008 | 937 | 1929241 |
| U_aymondiana_196_6 | 4695 | 4428 | 3577 | 2321898 |
| U_aymondiana_197_1 | 1631 | 1598 | 1498 | 2209716 |
| U_ciliata_287_1 | 7372 | 6127 | 4012 | 2315559 |
| U_ciliata_3773 | 5606 | 4801 | 3543 | 561139 |
| U_ciliata_3777 | 6609 | 5561 | 3860 | 709070 |
| U_ciliata_3829 | 11397 | 7382 | 4219 | 2049511 |
| U_ciliata_3840 | 11723 | 7440 | 4235 | 1582820 |
| U_ciliata_3845 | 10770 | 7471 | 4255 | 2318879 |
| U_ciliata_N27 | 8340 | 6169 | 3806 | 943652 |
| U_ciliata_N29 | 8263 | 6407 | 3970 | 1181793 |
| U_ciliata_New23 | 5423 | 4705 | 3472 | 993062 |
| U_ciliata_New24 | 6439 | 5509 | 3843 | 1120510 |
| U_ciliata_New6 | 2674 | 2435 | 2136 | 1367348 |
| U_ciliata_New8 | 3741 | 3339 | 2791 | 1357349 |
| U_fibriloides_197_3 | 1249 | 1211 | 1140 | 3134119 |
| U_fibriloides_197_4 | 4411 | 4197 | 3482 | 1997158 |
| U_hyyppae_4013 | 8435 | 7110 | 4325 | 2871881 |
| U_hyyppae_4070 | 7811 | 6865 | 4313 | 7209232 |
| U_hyyppae_4131 | 8656 | 7376 | 4341 | 1909073 |
| U_hyyppae_4132 | 7838 | 6927 | 4303 | 2402641 |
| U_hyyppae_4133 | 9509 | 7752 | 4442 | 5676264 |
| U_hyyppae_4143 | 5740 | 5306 | 3884 | 7069523 |
| U_hyyppae_4158 | 7540 | 6669 | 4250 | 6138554 |
| U_sp_N83 | 7872 | 6412 | 4010 | 1277105 |
| U_sp_New35 | 4878 | 4185 | 2981 | 1051124 |
| U_sp_New49 | 6511 | 5537 | 3867 | 1196396 |
| U_intermedia_FG656 | 7246 | 5380 | 3563 | 1252426 |
| U_intermedia_FG673 | 11570 | 6855 | 3969 | 4163774 |
| U_lambii_166_3 | 3834 | 3573 | 3022 | 2541753 |
| U_lambii_221_3a | 1708 | 1622 | 1470 | 2309829 |
| U_lambii_N111 | 8408 | 7144 | 4294 | 1427016 |
| U_lambii_N92 | 9205 | 7405 | 4295 | 1175689 |
| U_messutiae_209_1 | 4653 | 4342 | 3568 | 1911426 |
| U_messutiae_209_3 | 5452 | 5013 | 3879 | 2054045 |
| U_messutiae_41 | 2140 | 2038 | 1846 | 1556583 |
| U_pallidocarpa_193_11 | 6821 | 6139 | 4212 | 1903871 |
| U_pallidocarpa_193_7 | 6916 | 6101 | 4177 | 1836473 |
| U_pallidocarpa_210_3 | 5679 | 5249 | 3974 | 1452957 |
| U_patagonica_195_1 | 1580 | 1443 | 1290 | 3531961 |
| U_patagonica_266_5 | 4065 | 3605 | 2950 | 1968664 |
| U_patagonica_63 | 2022 | 1816 | 1600 | 1040961 |
| U_perplexans_FG693_B | 11488 | 6631 | 3899 | 3374597 |
| U_perplexans_FG706_A | 12096 | 6965 | 4016 | 3750434 |
| U_perpusilla_208_19 | 1864 | 1810 | 1675 | 1705539 |
| U_perpusilla_221_2 | 2488 | 2352 | 2096 | 2849100 |
| U_perpusilla_221_4 | 4181 | 3948 | 3310 | 1814284 |
| U_perpusilla_222_2 | 2682 | 2537 | 2142 | 3307275 |
| U_perpusilla_227_4 | 3685 | 3462 | 3003 | 2206310 |
| U_pseudocapillaris_3758 | 5384 | 4656 | 3452 | 724274 |
| U_pseudocapillaris_3760 | 4971 | 4374 | 3386 | 625551 |
| U_pseudocapillaris_3802 | 4553 | 3999 | 3178 | 754889 |
| U_pseudocapillaris_3837 | 9319 | 7030 | 4154 | 2364459 |
| U_pseudocapillaris_N77 | 2087 | 1881 | 1635 | 1122008 |
| U_pseudocapillaris_N79 | 5915 | 5000 | 3621 | 1177224 |
| U_pseudocapillaris_New45 | 1783 | 1643 | 1459 | 1165090 |
| U_rohmederi_218_1 | 3658 | 3433 | 2898 | 1299539 |
| U_rohmederi_218_3 | 5130 | 4703 | 3641 | 1417539 |
| U_rohmederi_221_3b | 3450 | 3226 | 2815 | 3288711 |
| U_rohmederi_227_1 | 3266 | 3059 | 2682 | 2239178 |
| U_rohmederi_227_5 | 6463 | 5782 | 4049 | 3043835 |
| U_trachycarpa_18684 | 12028 | 8044 | 4399 | 1642433 |
| U_trachycarpa_18714 | 8765 | 7225 | 4302 | 1843294 |
| U_trachycarpa_18724 | 11200 | 7816 | 4381 | 1701232 |
| U_trachycarpa_18803 | 11296 | 8019 | 4396 | 2064865 |
| U_trachycarpa_18804 | 11332 | 8013 | 4407 | 1825383 |
| U_trachycarpa_19072 | 9802 | 7589 | 4354 | 1883978 |
| U_sphacelata_220_3 | 4907 | 4534 | 3560 | 1417807 |
| U_sphacelata_226_1 | 1668 | 1604 | 1467 | 2910550 |
| U_sphacelata_243_3 | 2385 | 2269 | 2043 | 1064583 |
| U_sphacelata_N107 | 1708 | 1615 | 1448 | 1314759 |
| U_sphacelata_N110 | 2351 | 2231 | 2014 | 1115969 |
| U_sphacelata_N526_1 | 1296 | 1242 | 1122 | 1343717 |
| U_sphacelata_New43 | 1839 | 1761 | 1593 | 1412152 |
| U_sphacelata_New51 | 1338 | 1290 | 1183 | 1409658 |
| U_subantarctica_165_2 | 2189 | 2110 | 1929 | 1300758 |
| U_subantarctica_203_1 | 1294 | 1252 | 1148 | 1562032 |
| U_subantarctica_203_11 | 1100 | 1050 | 969 | 2035203 |
| U_subantarctica_264_1 | 1971 | 1897 | 1729 | 1487404 |
| U_subcapillaris_283_1 | 1435 | 1337 | 1195 | 2090317 |
| U_subcapillaris_284_1 | 2764 | 2526 | 2210 | 1722932 |
| U_subcapillaris_286_1 | 5711 | 4951 | 3753 | 2172897 |
| U_subcapillaris_N100 | 3957 | 3534 | 2802 | 1250710 |
| U_subcapillaris_N108 | 3088 | 2735 | 2193 | 1283035 |
| U_subcapillaris_N12 | 1231 | 1148 | 1012 | 1401994 |
| U_subcapillaris_N204 | 4874 | 4195 | 3144 | 1224067 |
| U_subcapillaris_N41 | 9162 | 7049 | 4195 | 1320080 |
| U_subcapillaris_New32 | 4168 | 3680 | 2893 | 932585 |
| U_subcapillaris_New48 | 3050 | 2766 | 2353 | 1107232 |
| U_subfloridana_702A1 | 7331 | 5545 | 3654 | 1310135 |
| U_subfloridana_717B2 | 7488 | 5562 | 3647 | 1119711 |
| U_taylorii_20812 | 5915 | 5344 | 3930 | 1945014 |
| U_taylorii_21003 | 9060 | 7145 | 4200 | 1639400 |
| U_taylorii_20877 | 4155 | 3763 | 2977 | 2182626 |
| U_trachycarpoides_173_1 | 3650 | 3489 | 3010 | 2265461 |
| U_trachycarpoides_182_3 | 1897 | 1848 | 1731 | 2262616 |
| U_trachycarpoides_234_8 | 3745 | 3588 | 3131 | 1858954 |
| U_ushuaiensis_179_1 | 6370 | 5795 | 4069 | 1499049 |
| U_ushuaiensis_179_4 | 4153 | 3884 | 3306 | 1437076 |
| U_ushuaiensis_4034 | 8055 | 6990 | 4275 | 1779863 |
| U_ushuaiensis_4102 | 9183 | 7472 | 4309 | 1974466 |
| **Mean** | 5917.376 | 4705.896 | 3180.304 | 2179387.79 |
| **Standard Deviation** | 3619.83109939249 | 2340.84340612524 | 1141.65229173589 |  |
| **Minimum** | 938 | 804 | 706 |  |
| **Maximum** | 15073 | 8182 | 4442 |  |

**Table S4.** Chemical substances obtained through HPTLC analysis and chemotypes assigned for the species of taxonomic interests (clades A-D). ‘x’ indicates presence in high concentration and ‘±’ indicate traces.

| Clade | Species | Sample | Usnic | Norstistic | Salazinic | Consalazinic | Psoromic | Protocetraric | Squamatic | Gyrophoric | Hypostrepsilic | Neuropogolic | Placodiolic | Fatty acids | Chemotype |
| --- | --- | --- | --- | --- | --- | --- | --- | --- | --- | --- | --- | --- | --- | --- | --- |
| A | *U. ciliata* | N29 | x | x | x | x |  |  |  |  |  |  |  |  | Chem. 5 |
| A | *U. ciliata* | N27 | x | x | x | x |  |  |  |  |  |  |  |  | Chem. 5 |
| A | *U. ciliata* | New8 | x | x | x | x |  |  |  |  |  |  |  |  | Chem. 5 |
| A | *U. ciliata* | New6 | x | x | x | x |  |  |  |  |  |  |  |  | Chem. 5 |
| A | *U. ciliata* | 3845 | x | x | ± | ± |  |  |  |  |  |  |  |  | Chem. 5 |
| A | *U. ciliata* | 3840 | x | x | x | x | x |  |  |  |  |  |  |  | Chem. 2 |
| A | *U. ciliata* | 3829 | x |  |  |  | x |  |  |  |  |  |  |  | Chem. 4 |
| A | *U. ciliata* | 287-1 | x | x | x | x | x |  |  |  |  |  |  |  | Chem. 2 |
| A | *U. ciliata* | 3777 | x | x | x | x | x | X |  |  |  |  |  |  | Chem. 3 |
| A | *U. ciliata* | 3773 | x | x | x | x | x | X |  |  |  |  |  |  | Chem. 3 |
| A | *U. ciliata* | New24 | x | x |  |  |  |  |  |  |  |  |  |  | Chem. 1 |
| A | *U. ciliata* | New23 | x | x | x | x |  |  |  |  |  |  |  |  | Chem. 5 |
| A | *U. subcapillaris* | New32 | x | x | x | x | x | ± |  |  |  |  |  |  | Chem. 6 |
| A | *U. subcapillaris* | New48 | x | x | x | x | x | ± |  |  |  |  |  |  | Chem. 6 |
| A | *U. subcapillaris* | N41 | x | x | x | x | x | ± |  |  |  |  |  |  | Chem. 6 |
| A | *U. subcapillaris* | 286-1 | x | x | x | x |  | ± | x |  |  |  |  |  | Chem. 5 |
| A | *U. subcapillaris* | 283-1 | x |  |  |  |  |  | x |  |  |  |  |  | Chem. 1 |
| A | *U. subcapillaris* | N204 | x | ± | x | ± |  | ± |  |  |  |  |  |  | Chem. 6 |
| A | *U. subcapillaris* | N12 | x |  |  |  | ± | ± | x |  |  |  |  |  | Chem. 4 |
| A | *U. subcapillaris* | 284-1 | x | x | x | x | ± |  |  |  |  |  |  |  | Chem. 3 |
| A | *U. subcapillaris* | N100 | x |  |  | x |  |  | x |  |  |  |  |  | Chem. 2 |
| A | *U. subcapillaris* | N108 | x |  |  | x |  |  | x |  |  |  |  |  | Chem. 2 |
| A | *U. sp.* | New49 | x | x | x | x | x |  |  |  |  |  |  |  | Chem. 1 |
| A | *U. sp.* | New35 | x | x | x | x | x |  |  |  |  |  |  |  | Chem. 1 |
| A | *U. sp.* | N83 | x | x | x | x | x |  |  |  |  |  |  |  | Chem. 1 |
| A | *U. pseudocapillaris* | 3760 | x | x | x | x | ± |  |  |  |  |  |  |  | Chem. 1 |
| A | *U. pseudocapillaris* | 3758 | x | x | x | x | ± |  |  |  |  |  |  |  | Chem. 1 |
| A | *U. pseudocapillaris* | 3802 | x | x | x | x | ± |  |  |  |  |  |  |  | Chem. 1 |
| A | *U. pseudocapillaris* | 3837 | x | x | ± | ± |  |  |  |  |  |  |  |  | Chem. 2 |
| A | *U. pseudocapillaris* | New45 | x | x | x | x | x |  |  |  |  |  |  |  | Chem. 1 |
| A | *U. pseudocapillaris* | N77 | x | x | x | x |  |  |  |  |  |  |  |  | Chem. 2 |
| A | *U. pseudocapillaris* | N79 | x | x | x | x |  |  |  |  |  |  |  |  | Chem. 2 |
| B | *U. rohmederi* | 221-3b | x |  |  |  |  |  |  |  |  |  |  |  | Chem. 1 |
| B | *U. rohmederi* | 227-1 | x |  |  |  |  |  |  |  |  |  |  |  | Chem. 1 |
| B | *U. rohmederi* | 218-1 | x |  |  |  |  |  |  |  |  |  |  |  | Chem. 1 |
| B | *U. rohmederi* | 218-3 | x |  |  |  |  |  |  |  |  |  |  |  | Chem. 1 |
| B | *U. rohmederi* | 227-5 | x |  |  |  |  |  |  |  |  |  |  |  | Chem. 1 |
| C | *U. perpusilla* | 208-19 | x |  |  |  | ± |  |  |  |  |  |  |  | Chem. 2 |
| C | *U. perpusilla* | 222-2 | x |  |  |  |  |  |  |  |  |  |  |  | Chem. 1 |
| C | *U. perpusilla* | 221-4 | x |  |  |  | ± |  |  |  |  |  |  |  | Chem. 2 |
| C | *U. perpusilla* | 221-2 | x |  |  |  |  |  |  |  |  |  |  |  | Chem. 1 |
| C | *U. perpusilla* | 227-4 | x |  |  |  | ± |  |  |  |  |  |  |  | Chem. 2 |
| D | *U. aymondiana* | 196-6 | x |  |  |  |  | ± |  |  |  |  |  | x | Chem. 1 |
| D | *U. aymondiana* | 197-1 | x |  |  |  |  | ± |  |  |  |  |  | x | Chem. 1 |
| D | *U. aymondiana* | 7-186-9 | x |  |  |  | x |  |  |  |  |  |  | x | Chem. 2 |
| D | *U. fibriloides* | 197-4 | x | x | x | x |  |  |  |  |  |  |  |  | Chem. 1 |
| D | *U. fibriloides* | 197-3 | x | x | x | x |  |  |  |  |  |  |  |  | Chem. 1 |
| D | *U. sphacelata* | 220-3 | x |  |  |  |  |  |  |  |  |  |  |  | Chem. 1 |
| D | *U. sphacelata* | 226-1 | x |  |  |  |  |  |  |  |  |  |  |  | Chem. 1 |
| D | *U. sphacelata* | New51 | x |  |  |  |  |  |  |  |  |  |  |  | Chem. 1 |
| D | *U. sphacelata* | N526-1 | x |  |  |  |  |  |  |  |  |  |  |  | Chem. 1 |
| D | *U. sphacelata* | N107 | x |  |  |  |  |  |  |  |  |  |  |  | Chem. 1 |
| D | *U. sphacelata* | N110 | x |  |  |  | ± |  |  |  |  |  |  |  | Chem. 2 |
| D | *U. trachycarpa* | 18804 | x | x | x | x | x |  |  |  |  |  |  | ± | Chem. 1 |
| D | *U. trachycarpa* | 18803 | x | x | +- |  | x |  |  | ± | ± |  | ± | x (muronic acid) | Chem. 2 |
| D | *U. trachycarpa* | 19072 | x | x |  |  |  |  |  | ± | ± |  | ± | x (muronic acid) | Chem. 3 |
| D | *U. trachycarpa* | 18714 | x |  |  |  |  |  |  |  | ± |  |  | x (muronic acid) | Chem. 4 |
| D | *U. trachycarpa* | 18724 | x | x |  |  |  |  |  |  |  |  | ± | x (muronic acid) | Chem. 5 |
| D | *U. trachycarpa* | 18684 | x |  |  |  |  |  | x?? |  |  | x | ± | ± | Chem. 6 |
| D | *U. subantarctica* | 165-2 | x |  |  |  |  |  |  |  |  |  |  |  | Chem. 1 |
| D | *U. subantarctica* | 203-11 | x | x |  |  |  |  |  |  |  |  |  |  | Chem. 2 |
| D | *U. subantarctica* | 264-11 | x | x | ± | ± |  |  |  |  |  |  |  |  | Chem. 2 |
| D | *U. subantarctica* | 203-1 | x | x | ± |  |  |  |  |  |  |  |  |  | Chem. 2 |
| D | *U. trachycarpoides* | 182-3 | x | x |  |  |  |  |  |  |  |  |  |  | Chem. 1 |
| D | *U. trachycarpoides* | 173-1 | x | x | x | x |  |  |  |  |  |  |  |  | Chem. 2 |
| D | *U. trachycarpoides* | 234-8 | x | x | x | x |  |  |  |  |  |  |  |  | Chem. 2 |
| D | *U. hyyppae* | 4131 | x |  |  |  | ± | ± |  |  |  |  |  |  | Chem. 1 |
| D | *U. hyyppae* | 4132 | x |  |  |  | ± |  |  |  |  |  |  |  | Chem. 1 |
| D | *U. hyyppae* | 4133 | x |  |  |  | ± |  |  |  |  |  |  |  | Chem. 1 |
| D | *U. hyyppae* | 4013 | x | x |  |  | ± |  |  |  |  |  |  |  | Chem. 2 |
| D | *U. hyyppae* | 4143 | x | x |  |  | ± |  |  |  |  |  |  |  | Chem. 2 |
| D | *U. hyyppae* | 4158 | x |  |  |  | ± |  |  |  |  |  |  |  | Chem. 1 |
| D | *U. hyyppae* | 4070 | x |  |  |  |  |  |  |  |  |  |  |  | Chem. 1 |
